# High-throughput and targeted drug screens identify pharmacological candidates against MiT-translocation renal cell carcinoma

**DOI:** 10.1186/s13046-023-02667-4

**Published:** 2023-04-25

**Authors:** Martin Lang, Laura S. Schmidt, Kelli M. Wilson, Christopher J. Ricketts, Carole Sourbier, Cathy D. Vocke, Darmood Wei, Daniel R. Crooks, Youfeng Yang, Benjamin K. Gibbs, Xiaohu Zhang, Carleen Klumpp-Thomas, Lu Chen, Rajarshi Guha, Marc Ferrer, Crystal McKnight, Zina Itkin, Darawalee Wangsa, Danny Wangsa, Amy James, Simone Difilippantonio, Baktir Karim, Francisco Morís, Thomas Ried, Maria J. Merino, Ramaprasad Srinivasan, Craig J. Thomas, W. Marston Linehan

**Affiliations:** 1 Urologic Oncology Branch, Center for Cancer Research, National Cancer Institute, National Institutes of Health, Bethesda, MD USA; 2 Institute for Biomedicine, Eurac Research, Affiliated Institute of the University of Lübeck, Bolzano, 39100 Italy; 3 Basic Science Program, Frederick National Laboratory for Cancer Research, Frederick, MD USA; 4 Division of Preclinical Innovation, National Center for Advancing Translational Sciences (NCATS), Bethesda, MD USA; 5 Genetics Branch, Cancer Genomics Section, Center for Cancer Research, National Cancer Institute, National Institutes of Health, Bethesda, MD USA; 6 Laboratory of Animal Sciences Program, Frederick National Laboratory for Cancer Research, Frederick, MD USA; 7 EntreChem SL, Vivero Ciencias de la Salud, Calle Colegio Santo Domingo Guzmán, Oviedo, AS 33011 Spain; 8 Laboratory of Pathology, National Cancer Institute, National Institutes of Health, Bethesda, MD USA

**Keywords:** MiT family translocation RCC, TFE3-RCC, TFE3-fusion, GPNMB, RCC therapy, NVP-BGT226, Mithramycin A, CDX-011

## Abstract

**Background:**

MiT-Renal Cell Carcinoma (RCC) is characterized by genomic translocations involving microphthalmia-associated transcription factor (MiT) family members *TFE3*, *TFEB*, or *MITF*. MiT-RCC represents a specific subtype of sporadic RCC that is predominantly seen in young patients and can present with heterogeneous histological features making diagnosis challenging. Moreover, the disease biology of this aggressive cancer is poorly understood and there is no accepted standard of care therapy for patients with advanced disease. Tumor-derived cell lines have been established from human TFE3-RCC providing useful models for preclinical studies.

**Methods:**

TFE3-RCC tumor derived cell lines and their tissues of origin were characterized by IHC and gene expression analyses. An unbiased high-throughput drug screen was performed to identify novel therapeutic agents for treatment of MiT-RCC. Potential therapeutic candidates were validated in in vitro and in vivo preclinical studies. Mechanistic assays were conducted to confirm the on-target effects of drugs.

**Results:**

The results of a high-throughput small molecule drug screen utilizing three TFE3-RCC tumor-derived cell lines identified five classes of agents with potential pharmacological efficacy, including inhibitors of phosphoinositide-3-kinase (PI3K) and mechanistic target of rapamycin (mTOR), and several additional agents, including the transcription inhibitor Mithramycin A. Upregulation of the cell surface marker *GPNMB*, a specific MiT transcriptional target, was confirmed in TFE3-RCC and evaluated as a therapeutic target using the GPNMB-targeted antibody-drug conjugate CDX-011. In vitro and in vivo preclinical studies demonstrated efficacy of the PI3K/mTOR inhibitor NVP-BGT226, Mithramycin A, and CDX-011 as potential therapeutic options for treating advanced MiT-RCC as single agents or in combination.

**Conclusions:**

The results of the high-throughput drug screen and validation studies in TFE3-RCC tumor-derived cell lines have provided in vitro and in vivo preclinical data supporting the efficacy of the PI3K/mTOR inhibitor NVP-BGT226, the transcription inhibitor Mithramycin A, and GPNMB-targeted antibody-drug conjugate CDX-011 as potential therapeutic options for treating advanced MiT-RCC. The findings presented here should provide the basis for designing future clinical trials for patients with MiT-driven RCC.

**Supplementary Information:**

The online version contains supplementary material available at 10.1186/s13046-023-02667-4.

## Background

Renal cell carcinoma (RCC), one of the ten most common cancers, occurs in both sporadic and inherited forms. MiT family translocation RCC, is an aggressive form of the disease [1–3] which accounts for 1–5% of sporadic RCC and represents 42% of kidney cancer in children and young adults [4]. MiT family translocation RCC is characterized by gene fusions resulting from somatic chromosomal translocations involving microphthalmia-associated transcription factor (MiT) family members TFE3, TFEB or MITF. These proteins are basic helix-loop-helix leucine zipper transcription factors that undergo homo- or hetero-dimerization and then bind to E-box sequences in target genes to promote transcription. MiT proteins are considered master regulators of lysosomal biogenesis, with additional diverse and tissue specific functions often related to cell growth and differentiation [5].

TFE3-fusion RCC, the most common form of MiT translocation RCC, is characterized by Xp11.2 chromosomal translocations involving TFE3 that result in a fusion of the C-terminal portion of TFE3 with the N-terminal portion of specific partner genes. Consequently, activity of the TFE3 C-terminus is dysregulated resulting in constitutive nuclear localization and increased transcriptional activity of the protein. The most common Xp11.2 translocation is t(X;1)(p11.2;q21), resulting in a *PRCC–TFE3* fusion protein [6]; other rearrangements include t(X;1)(p11.2;p34) leading to *SFPQ*–*TFE3* fusion, inv(X)(p11.2;q12) leading to *NONO*–*TFE3* fusion, t(X;17)(p11;q25) leading to *ASPSCR1*–*TFE3* fusion, and t(X;17)(p11;q23) leading to *CLCT*–*TFE3* fusion [5].

MiT translocation RCC is characterized by heterogeneous architectural and cytologic features that overlap with clear cell and papillary RCC as well as oncocytic tumors [7], and have historically been frequently underdiagnosed. Recent TCGA data confirmed that MiT translocation RCC is more common than appreciated and is found in up to 12% of type 2 papillary RCC in adult patients [8].

TFE3-fusion RCC is a highly aggressive form of renal cancer and affected patients often present with metastasis at initial diagnosis. There is currently no clinical standard of effective systemic treatment and no effective targeted drug therapies have been identified to date. Activity of tyrosine kinase inhibitors (TKI) against TFE3-RCC has recently been reported in in vitro organoid-based experiments [9], and a clinically complete response to the multi-targeted TKI sunitinib was reported in a child with metastatic *TFE3* translocation RCC [10]. A few retrospective studies of adult patients with metastatic TFE3 fusion RCC have reported incomplete responses to TKIs and mTOR inhibitors [11, 12]. Despite genomics or transcriptomics analyses of TFE3-fusion RCC, information leading to an effective therapeutic approach targeting key signaling pathways that drive these tumors is lacking [13, 14].

A known biomarker and potential therapeutic target for TFE3-fusion RCC is glycoprotein nonmetastatic melanoma B (GPNMB), a highly glycosylated transmembrane protein that regulates a variety of physiological processes, notably osteoclast differentiation and melanosome maturation [15]. *GPNMB* is overexpressed in a number of cancer types [16, 17] and was first shown to be transcriptionally regulated by MITF [18, 19], but subsequently was identified as a direct transcriptional target of TFE3 and is highly expressed in TFE3-fusion RCC [20, 21].

Unbiased high-throughput drug screens are a potential first line approach to identifying key signaling pathways for the development of therapies. Herein we have conducted quantitative high-throughput screens of multiple TFE3-fusion RCC cell lines derived from TFE3-fusion renal tumors and identified classes of drugs with cytotoxicity against TFE3-fusion RCC. We further validated the cytotoxicity and increased apoptosis of selected agents from each class and their effects on cell cycle and downstream pathway members in 2D and 3D in vitro assays and TFE3-fusion derived xenograft models. Notably, we identified the TFE3 transcriptional target GPNMB as a cell surface biomarker that is upregulated in TFE3-fusion RCC, and evaluated a clinically relevant antibody-drug conjugate (ADC) that targets this protein in in vitro and in vivo assays. Finally, combinations of these drugs with the ADC were assessed for potential synergy as therapeutic agents in the TFE3-fusion cell lines and xenograft models.

## Materials and methods

### Patients

Patients were seen at the Urologic Oncology Branch (UOB) of the National Cancer Institute (NCI), National Institutes of Health (NIH) for clinical assessment. This study was approved by the Institutional Review Board of the National Cancer Institute and patients provided written informed consent on either Urologic Oncology Branch protocol NCI-89-C-0086 or NCI-97-C-0147.

### Cell lines and cell culture

The TFE3-fusion RCC cell lines UOK109, UOK120, UOK124, UOK145, and UOK146 were developed within the Urologic Oncology Branch (NCI) from surgically resected specimens (2, 22, 23). The clear cell RCC-derived UOK140 cell line (22) was used as negative control in CDX-011 experiments. Cells were maintained at 37ºC with 5% CO_2_ and were cultured in high glucose (4.5g/l) Dulbecco’s modified Eagle’s medium (DMEM; Gibco, Gaithersburg, MD) supplemented with L-glutamine (4 mM), sodium pyruvate (110mg/l), and 10% fetal bovine serum (Sigma Aldrich, St. Luis, MO).

### Sanger sequencing

DNA was extracted from tumor tissue as above using the Maxwell Tissue Kit (Promega, WI, USA). PCR reactions to amplify the sequences across the gene fusions were performed using KAPA2G Fast DNA polymerase (Roche, Indianapolis, IN) according to the manufacturer’s specifications. Bidirectional Sanger DNA sequencing of the PCR products was performed using the Big Dye Terminator v.1.1 Cycle Sequencing Kit (Applied Biosystems, CA, USA) according to the manufacturer’s specifications and run on an ABI 3130xl or 3730 Genetic Analyzer (Applied Biosystems). Sanger Sequencing was conducted at the CCR Genomics Core at the National Cancer Institute, NIH, Bethesda, MD 20,892. Forward and reverse sequences were evaluated using Sequencher 5.0.1 (Genecodes, MI, USA). Fusion partner-specific primers to characterize the gene fusions were as follows: TFE3_Rv: GCAGGAGTTGCTGACAGTGA; NONO_Fw: ATCAAGGAGGCTCGTGAG; PRCC_Fw1: AGGAAAGAGCCCGTGAAGAT (UOK120, UOK146): PRCC_Fw2: ATGCCGCTGGTGCTTATTAT (UOK124); SFPQ_Fw: CTTTTGCGCCAAGATCTGA.

### Quantitative high-throughput screening

In collaboration with the National Center for Advancing Translational Sciences, NIH, quantitative high-throughput screening (qHTS) of 1912 pharmacologically defined small molecule compounds was conducted on three TFE3-fusion cell lines bearing three different fusion partners (UOK109, UOK124, UOK145) as outlined in Supplemental Methods.

### Cell viability assays

2D cell viability following 48–72 h drug treatment was evaluated using CellTiter-Glo reagent (Promega) according to manufacturer’s instructions. Assays were performed in triplicate and experiments were repeated three times in all five cell lines. Spheroids were successfully developed in three of the cell lines, UOK109, UOK120, and UOK124, using a previously described methodology [24] to evaluate 3D cell viability. Spheroids were incubated with drug for five days, before assessing viability by CellTiter-Glo 3D Cell Viability Assay (Promega). Calculation of drug synergy was performed with Compusyn [25], according to software instructions, including at least 6 concentration points for each drug alone and in combination. Cell cytotoxicity in vitro was measured with the lactate dehydrogenase (LDH)-based Cytotoxicity Detection Kit (Roche) as previously described [26].

### Reagents and resources

Commercially available reagents and resources included Mithramycin A (Tocris) and NVP-BGT226 (Selleck Chemicals). EC-8042 (EntreChem SL), antibody-drug conjugate CDX-011 and non-conjugated antibody CR011 (Celldex Therapeutics) were obtained from the manufacturers through collaborative agreements. All other compounds were generously provided by the NCI Development Therapeutics Program, NIH.

### Flow cytometry assays

Cell cycle and cell apoptosis analyses were performed by flow cytometry as previously described [27] using anti-cleaved PARP (BD Horizon) and anti-cleaved Caspase 3 (Cell Signaling Technologies) antibodies. Cell surface expression of GPNMB was measured by flow cytometry using anti-GPNMB (R&D Systems AF2550) or CR011 (Celldex Therapeutics) antibodies following 30-minute treatment with fixative solution or fixation/permeabilization solution (BD Biosciences). Cells were washed and resuspended in MACS buffer (PBS, 0.5% BSA, 2mM EDTA) for analysis. All samples were run on a BD FACS Canto II flow cytometer (Becton Dickinson, NJ) and analyzed with FlowJo Software (FlowJo, OR).

### SP1 transcription factor reporter assay

SP1 reporter assay was performed with Cignal Luciferase Reporter Assay Kit (Qiagen) following manufacturer’s instructions and outlined in Supplementary Methods.

### Immunoblots

Protein lysates were prepared from cell lines or frozen tissues using RIPA buffer (Thermo Fisher Scientific, Waltham, MA) supplemented with protease and phosphatase inhibitors (Thermo Fisher Scientific). Western Blot analyses were performed with standard techniques using the following antibodies: β-Actin, total Src, phosphoSrc (T416), phospho-ERK1/2 (T202/204), total and phospho-Akt (S473), total and phospho-S6 (S240/244), phospho-mTOR (S2448), phospho-4EBP1(T37/46), LC3B (all from Cell Signaling Technology), goat anti-human Osteoactivin/GPNMB antibody, p62 (both R&D Systems). Protein bands where visualized with IRDye secondary antibodies diluted in Odyssey blocking buffer containing 0.2% Tween and 0.1% SDS and signals were visualized with an Odyssey imager and analyzed with Image Studio software (all LI-COR Bioscience).

### Immunohistochemistry, FISH analysis, and spectral karyotyping

Hematoxylin and eosin staining was performed by standard methods. Histology was reviewed by a pathologist experienced in evaluating kidney cancer. Immunohistochemistry for TFE3 and GPNMB was performed as previously described [28]. Primary antibodies were as follows: Human Osteoactivin/GPNMB anti goat (R&D Systems; 1:800); TFE3 anti rabbit (Sigma Aldrich; 1:800). Spectral karyotyping and TFE3 was performed as previously described [29] and outlined in Supplementary Methods.

### Gene expression analysis by real-time-PCR

RNA was extracted from frozen tumor tissue and from cell lines with TRIzol™ Reagent (Thermo Fisher Scientific) according to manufacturer’s instructions and converted to cDNA with SuperScript IV VILO cDNA synthesis kit (Invitrogen). Gene expression was measured by Real-Time PCR using TaqMan® Gene Expression Assays on a ViiA7 Real-Time PCR system (Thermo Fisher Scientific) per manufacturer’s instructions. All assays were run in triplicate and gene expression was calculated as comparative CT (ΔΔCT) values. Gene expression was evaluated for *GPNMB* (Hs01095669_m1) and *BIRC5* (Hs04194392_s1), using *ACTB* (Hs01060665_g1) as reference gene.

### ***In Vivo *studies**

Tumor xenografts were generated by injecting UOK124 or UOK146 cells subcutaneously into flanks of athymic nude mice (Charles River). Mice were randomized into treatment groups (n = 10 mice/group) based on tumor volume and treated with the following agents, singly or in combination: (1) NVP-BGT226, (2) Mithramycin A, (3) Dasatinib, (4) Carfilzomib, (5) CDX-011, (6) respective vehicle. Details are outlined in Supplementary Methods. Body weights and tumor measurements were taken to determine response. Mouse survival was calculated as Log-rank test in Graphpad Prism and xenograft growth rates were compared based on rate-based T/C metric according to Hather et al. [30] and as outlined in Supplementary Methods.

### Statistical analysis

Values are expressed as mean ± standard deviation. Where appropriate, data were analyzed using a two-tailed *t* (parametric) test or Mann-Whitney (non-parametric) test, with a p value < 0.05 considered significant. All experiments were performed three times, with exception of the animal studies which were performed once using 2 cell lines.

## Results

### Description of TFE3 -fusion RCC cell line models

**Fig. 1:**
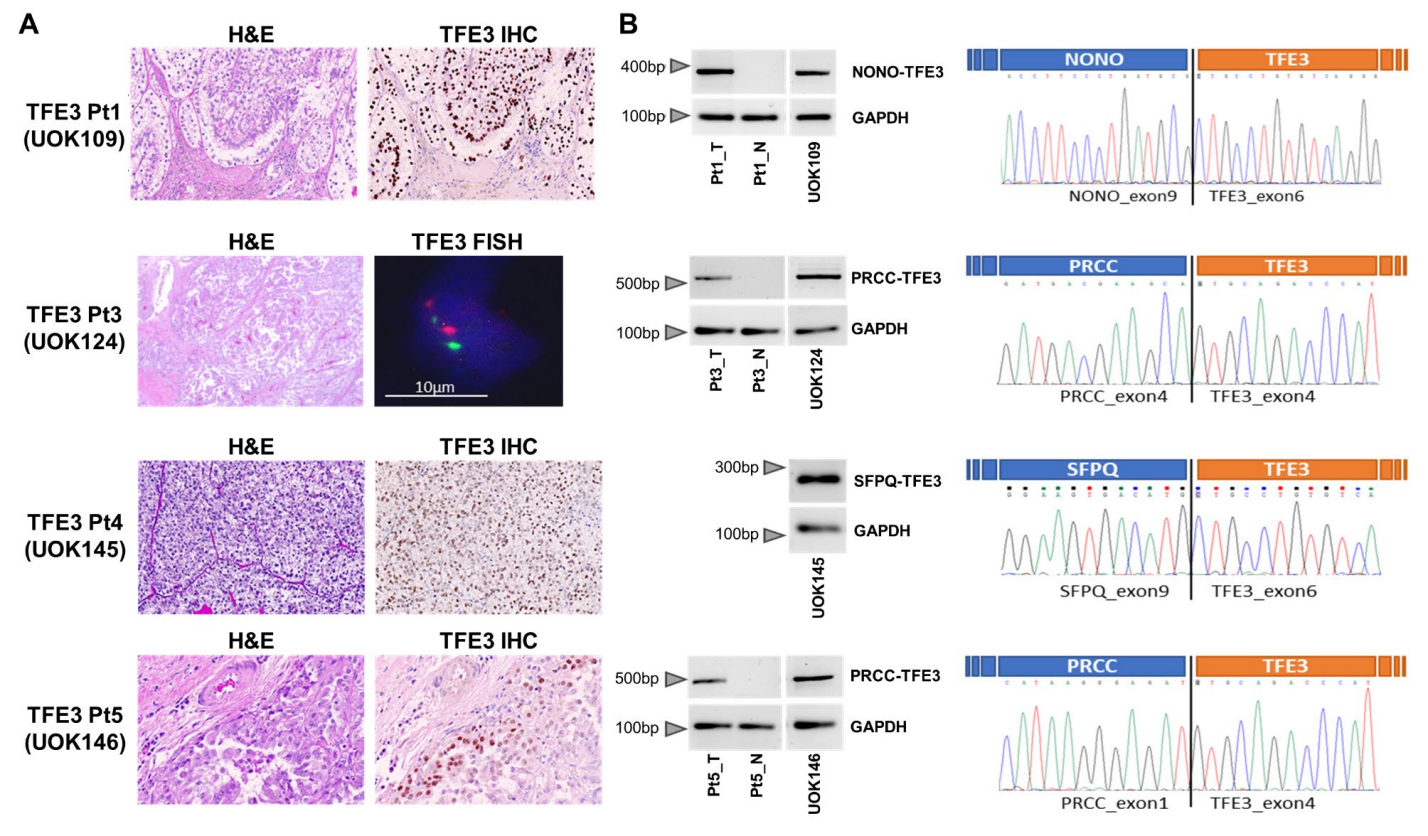
Histological and genetic characterization of MiT-driven RCC and TFE3-fusion RCC-derived cell lines. H&E staining of four representative TFE3-fusion tumors from which cell lines were generated (**A**) shows a typical growth pattern with papillary architecture and clear cells. Nuclear immunohistochemical staining of TFE3 is demonstrated in three TFE3-fusion tumors (100x) (**A**). Break-apart FISH for Patient 3 reveals nuclei with two separate fluorescent signals from TFE3 3’ and 5’ probes indicating translocation of chrX at the TFE3 locus (**A**). PCR amplification of *TFE3*-fusion genes and *GAPDH* controls was performed on cDNA from tumor tissues, corresponding normal kidney tissues, and four patient-derived TFE3-fusion RCC cell lines to confirm the presence of each gene fusion (**B**, left panels). Sanger sequencing electropherograms for cDNA spanning the breakpoint of each gene fusion are shown with genes and exons indicated (**B**, right panels)

We have previously established the five TFE3-fusion RCC cell lines included in this study (UOK109, UOK120, UOK124, UOK145 and UOK146) [2, 22, 23]. These cell lines were established from patients with an average age at surgery of 31.8 years. Additional phenotypic details are provided in Supplementary Table S1. TFE3 immunohistochemistry performed on the available formalin-fixed tumor tissues from which the cell lines were derived demonstrated strong nuclear staining, and break-apart fluorescent in situ hybridization (FISH) or karyotype analysis confirming the diagnosis in all samples (Fig. 1A). Tissue representing UOK120 was not available. Molecular characterization of the fusion genes was performed on frozen tumor material from three patients and four cell lines (Fig. 1B). Three cell lines carry *PRCC-TFE3* fusions (UOK120, UOK124, UOK146), one line is characterized by a *NONO-TFE3* fusion (UOK109) and one by an *SFPQ-TFE3* fusion (UOK145) (Fig. 1B, Supplementary Table S1). In addition to the distinguishing TFE3-fusion alteration, all lines carry further chromosomal alterations mainly leading to hyperdiploid karyotypes with modal chromosome numbers ranging from 49 to 74, as evidenced by spectral karyotyping (SKY) analysis (Supplementary Figure S1).

### Quantitative high-throughput screen (qHTS) of TFE3-fusion cell lines identifies potential therapeutic targets for TFE3-RCC

**Fig. 2:**
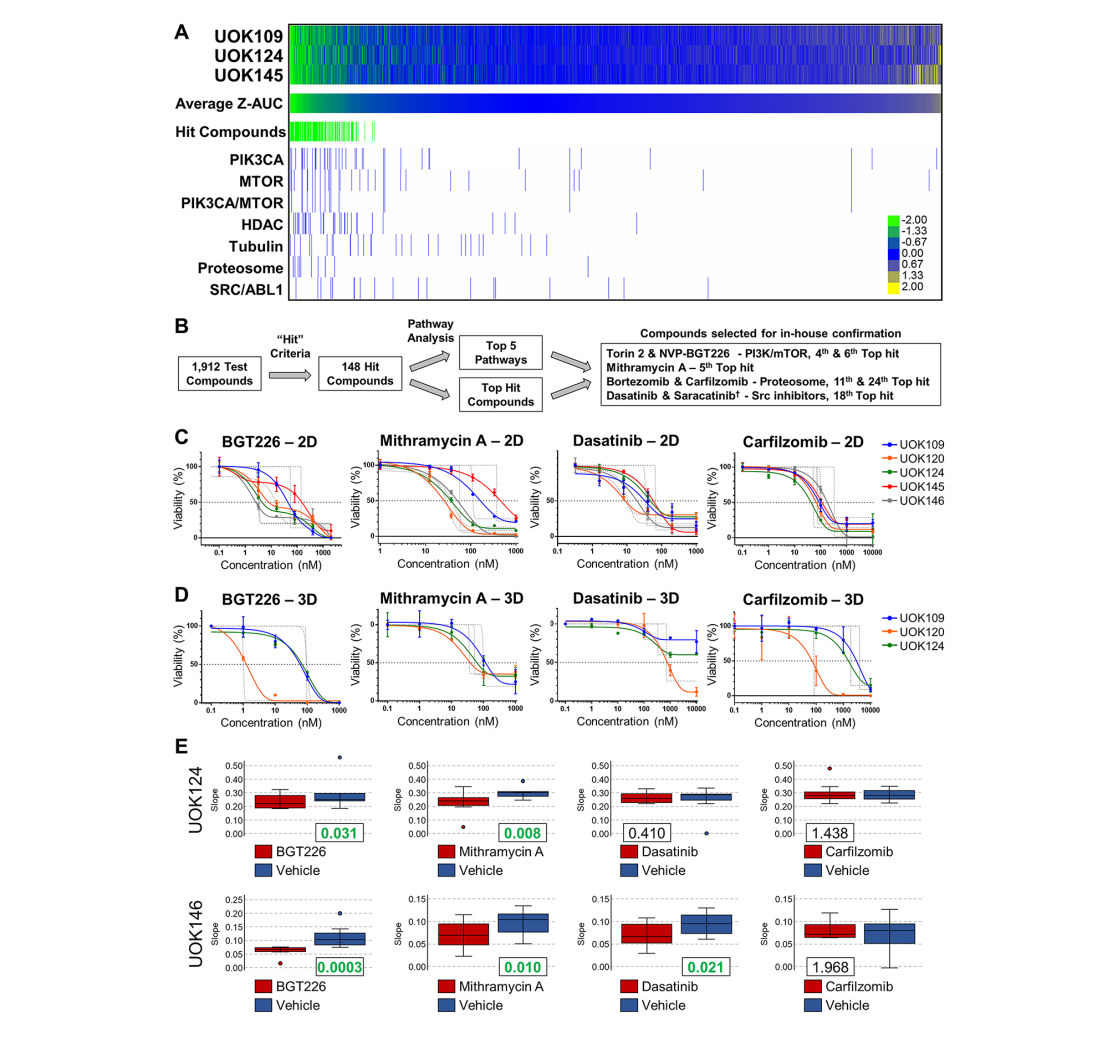
High-throughput screen and validation of pharmacological targets in vitro and in vivo. Heatmap shows viability as area under the curve (AUC) for compounds in the high-throughput small molecule drug screen using the Mechanism Interrogation PlatE (MIPE) library (**A**). The screen was performed on three cell lines with different TFE3-fusion partners (UOK109, UOK124, UOK145). Specific agents were selected from hit compounds (Z-AUC <-0.8 in all three cell lines) that were highly effective within these cell lines and represented enriched classes of agents from the pathway analysis (**B**). Viability curves in follow-up screens of TFE3-fusion cell lines in 2D (**C**) and 3D spheroid (**D**) growth conditions are shown. Drug candidates with confirmed significant effect on viability from high-throughput screen are displayed. Tumor growth rates calculated as a rate-based T/C (treated/control) metric for UOK124 and UOK146 mouse xenografts were determined for four representative drugs selected for the in vivo experiment, each of which was from a separate pharmacological class (**E**). Rate-based T/C ratio values are shown for each drug-vehicle pair. Significantly different values (< 0.4) are highlighted in bold green

The variable nature of the fusion partners and chromosomal alterations within the TFE3-fusion RCC tumors led us to perform a broad spectrum drug screen in 3 TFE3-fusion RCC cell lines (UOK109, UOK124, UOK145) with 3 different fusion partners to identify agents that would be effective across all fusions. In collaboration with the National Center for Advancing Translational Science (NCATS), a high-throughput drug screen was performed utilizing a library of 1912 pharmacologically defined small molecules with clinical relevance in cancer [31]. Hit compounds were identified that demonstrated an effect in all 3 cell lines and pathway analysis of these hit compounds highlighted enrichment for five classes of agents with potential pharmacological efficacy against the TFE3-fusion RCC cell lines, including inhibitors of the phosphoinositide-3-kinase (PI3K) / mechanistic target of rapamycin (mTOR) pathway, histone deacetylases (HDAC), tubulin, proteasome, and Src/Abl kinases (Fig. 2A,B). From this screen, the PI3K/mTOR inhibitors NVP-BGT226 and Torin 2, the proteasome inhibitors Carfilzomib and Bortezomib, and the Src inhibitors Dasatinib and Saracatinib were selected for validation by 2D viability assays in all 5 TFE3-fusion RCC cell lines (Fig. 2B,C). Concurrently, 3D viability assays were also performed in the 3 TFE3-fusion RCC cell lines that produced spheroids (UOK109, UOK120, UOK124) (Fig. 2D; Supplementary Figure S2). In addition, the RNA synthesis inhibitor Mithramycin A was selected for validation due to being one of the top 10 most effective compounds against UOK120, UOK124 and UOK145, the three cell lines in the high-throughput screen (Fig. 2A,C,D; Supplementary Figure S2). NVP-BGT226, Torin 2, Carfilzomib, Bortezomib, Dasatinib, and Mithramycin A caused reduced viability in both 2D and 3D assays, validating the qHTS results.

NVP-BGT226, Carfilzomib, Dasatinib, and Mithramycin A, representative agents from each drug class, were selected for further validation in mouse xenograft studies using the TFE3-fusion RCC cell lines UOK124 and UOK146 that consistently grew well as xenografts. Treatment with NVP-BGT226 and Mithramycin A caused significant tumor growth inhibition compared to vehicle controls in both xenograft models (Fig. 2E; Supplementary Figure S3A) and significantly increased survival (Supplementary Figure S3B) in UOK124 but not UOK146 xenografts. Dasatinib was effective in inhibiting tumor growth in UOK146, but not UOK124 xenografts, while efficacy of Carfilzomib was not significant in vivo (Fig. 2E; Supplementary Figure S3A). Only the mice bearing UOK146 tumors that were treated with Mithramycin A demonstrated any evidence of drug toxicity, showing a mild loss of mean body weight (Supplementary Figure S3C). Based on these data, NVP-BGT226, Mithramycin A, and Dasatinib were further investigated for their respective mechanisms of action against TFE3-fusion tumors.

### NVP-BGT226 targets the Akt/mTOR pathway, an important driver of TFE3-fusion RCC

**Fig. 3:**
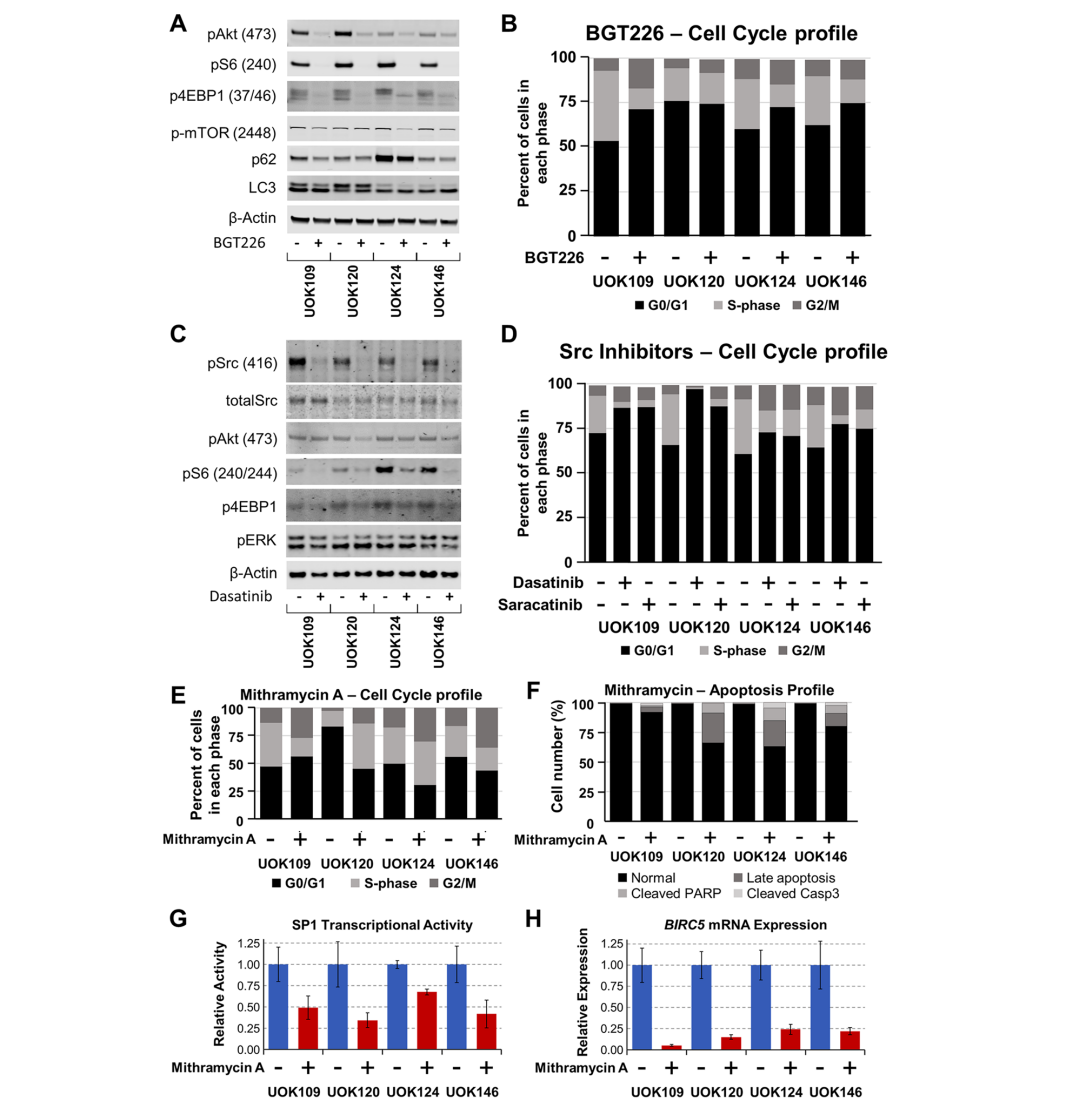
Mechanistic effects of NVP-BGT226, Dasatinib and Mithramycin A on TFE3-fusion RCC cell lines. Effect of NVP-BGT226 on activation of mTOR pathway in TFE3-fusion RCC cell lines as assessed by western blot measuring phosphorylation of downstream markers. Decrease in p62 levels and increase in LC3-II levels provide evidence of increased autophagy upon drug treatment (**A**). Summary breakdown of cell cycle analysis obtained by flow cytometry showing significant decrease of cells in cell cycle S-phase upon treatment with NVP-BGT226 (**B**). Western blot analysis of phospho-Src at Tyr416, phosphorylation of mTOR pathway targets Akt, S6 and 4EBP1, and MAPK pathway member ERK are shown for TFE3-fusion RCC cells treated for 24 h with Dasatinib (**C**). Proportion of Dasatinib and Saracatinib treated cells in a summary breakdown of cell cycle analysis are shown (**D**). Proportion of cells treated with Mithramycin A in a summary breakdown of cell cycle (**E**), and percentage of cells in late apoptosis phase (positive for cleaved PARP and cleaved Caspase 3) are shown (**F**). Transcriptional activity assay is presented as relative activity of SP1 transcription factor in treated vs. untreated cells (**G**). Gene expression of SP1 downstream target *BIRC5* is presented as relative amount of RNA in treated vs. untreated cells (**H**)

The efficacy of PI3K/mTOR inhibitors against TFE3-fusion RCC cell lines confirms the importance of the mTOR pathway as a driver of tumor cell growth (Fig. 2). To demonstrate the on-target effect of a PI3K/mTOR inhibitor, the phosphorylation of PI3K/mTOR signaling proteins Akt (Ser473), mTOR (Ser2448), S6 (Ser240), and 4EBP1 (Thr37/46) was shown by western blot to decrease in TFE3-fusion RCC cell lines after treatment with 100nM NVP-BGT226 (Fig. 3A; Supplementary Figure S4A). Similarly, drug treatment increased autophagy, as indicated by phosphatidylethanolamine conjugation of the microtubule-associated protein light chain 3 (LC3-II), and p62 degradation (Fig. 3A; Supplementary Figure S4A). In line with previous experiments evaluating inhibitors of mTOR in TFE3-fusion RCC cell lines [26, 31], NVP-BGT226 decreased the cell cycle S-phase, while not significantly inducing apoptosis (Fig. 3B; Supplementary Figure S4B,C).

Due to dose-limiting toxicities of NVP-BGT226 in clinical trials [32], we tested additional PI3K/mTOR inhibitors for their efficacy in the TFE3-fusion RCC cell lines and found that LY3023414 demonstrated an IC50 of 325-740nM in 2D studies and 622nM-3.25µM in 3D spheroid models (Supplementary Figure S2). Torin2, a potent novel dual mTOR complex inhibitor reduced growth of TFE3-fusion RCC cell lines in 2D and 3D models at low nanomolar concentrations (Supplementary Figure S2). Therefore, Torin2 presents an attractive therapeutic option against TFE3-fusion RCC.

### Src inhibition by Dasatinib decreases TFE3 tumor cell survival through the mTOR pathway

Given the significant effect of Dasatinib in the initial in vitro and in vivo studies (Fig. 2C-E, Supplementary Figure S2), we evaluated the pharmacological effect of Dasatinib on 2D cultures of TFE3-fusion RCC, and on Src activation and the activation of downstream pathway signaling proteins. Dasatinib decreased Src autophosphorylation and activation, and induced a decrease in Akt and mTOR target phosphorylation (S6, 4EBP1), but not ERK phosphorylation, suggesting that Src and at least one of its downstream pathways was inhibited by the treatment (Fig. 3C; Supplementary Figure S5A). In line with a primarily cytostatic effect in RCC cells [33], Dasatinib significantly decreased the cell cycle S phase in TFE3-fusion RCC cell lines (Fig. 3D; Supplementary Figure S5B). The inhibitory effect of the drug on the mTOR pathway may explain in part its effect on cell cycle and viability. While Dasatinib is less specific and can inhibit multiple kinases, the specific Src inhibitor Saracatinib showed inhibition of cell viability in 2D cultures (Supplementary Figure S2) and inhibition of the cell cycle S-phase (Fig. 3D; Supplementary Figure S5B). These data suggest an important role for Src kinase, in conjunction with other signaling pathways, in driving TFE3-fusion RCC tumor growth.

### **Mithramycin A inhibition of*BIRC5*(survivin) transcription affects TFE3-fusion RCC viability**

Since MiT-fusion RCC is driven by constitutively active transcription factors, the transcription inhibitor Mithramycin A (Plicamycin) represented a plausible pharmacological candidate. Notably, Mithramycin A scored high in the quantitative high-throughput screen. Additionally, TCGA gene expression data demonstrated that a known inhibition target of Mithramycin A, *BIRC5* (survivin), was highly upregulated in TFE3-fusion RCC (Supplementary Figure S6A). In our 2D and 3D in vitro models, Mithramycin A inhibited cell growth with EC50s of 28-333nM (Fig. 2C,D; Supplementary Figure S2A, S6B). The drug caused a blockage of the cell cycle at the G2/M phase (Fig. 3E; Supplementary Figure S6C) and induced marked apoptosis (Fig. 3F; Supplementary Figure S6D) at a concentration of 100nM. A transcriptional activity assay showed that Mithramycin A significantly decreased SP1 transcriptional activity in all TFE3-fusion RCC cell lines tested (Fig. 3G) and expression of the downstream target *BIRC5* was dramatically reduced upon drug treatment (Fig. 3H).

Mithramycin A shows relatively high toxicity in patients at therapeutic doses, and analogs with a better toxicity profile have been developed. We therefore tested the Mithramycin A analog with lowest reported toxicity, EC-8042 (EntreChem SL) [34], for its activity on TFE3-fusion RCC cells, and demonstrated an EC50 of 26-951nM paired with a minimum viability of ~ 10–65% (Supplementary Figure S2; Supplementary Figure S6E). Taken together, these data demonstrate that Mithramycin A and its low-toxicity analog inhibit signaling pathways important for TFE3-fusion RCC growth and survival and represent promising precision therapies against these tumors.

### A targeted drug study identified GPNMB as biomarker and therapeutic target of MiT RCC

**Fig. 4:**
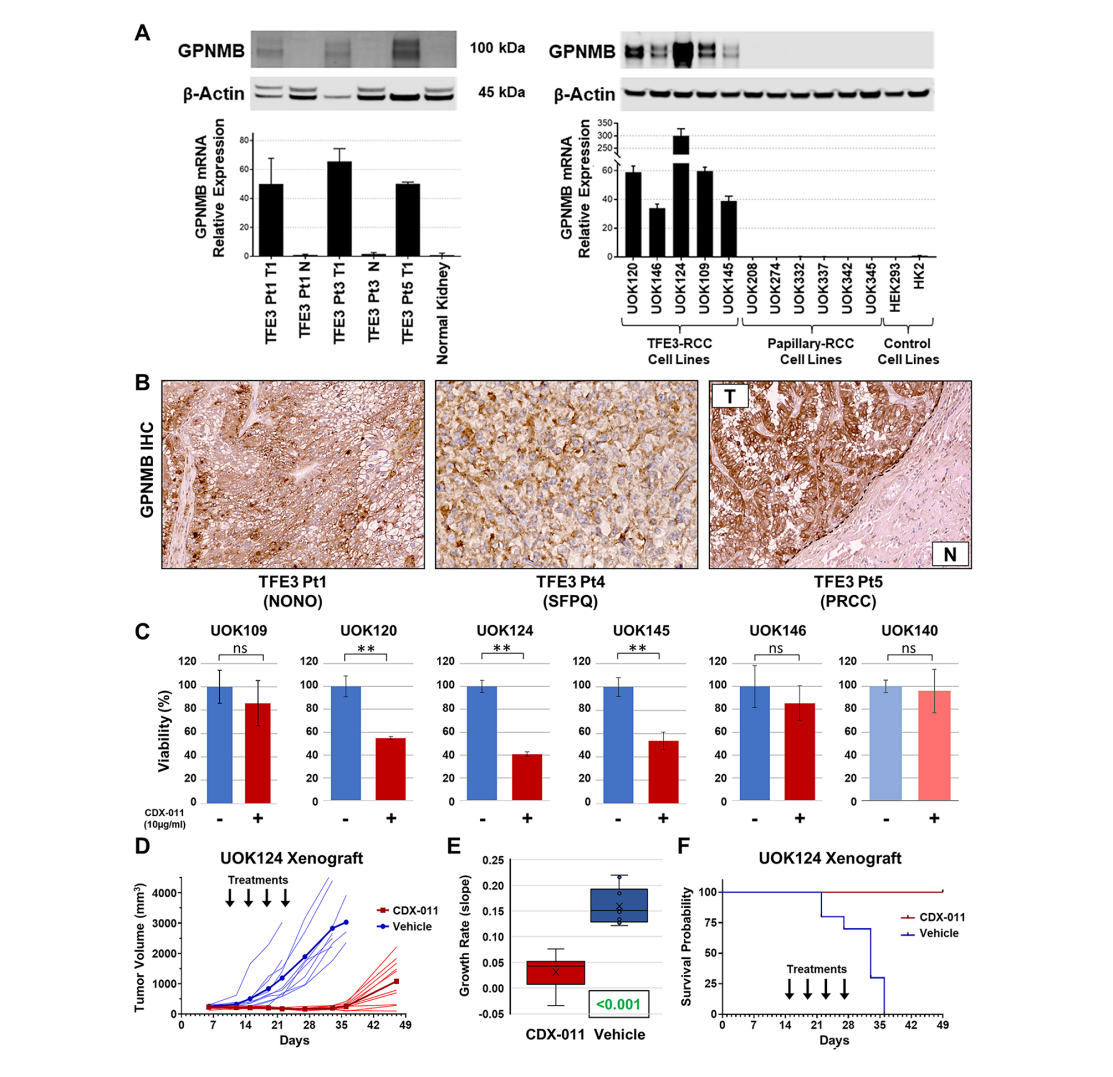
GPNMB, a diagnostic marker and therapeutic target of MiT-driven RCC. Expression of GPNMB at the mRNA and protein levels in three TFE3-fusion RCC tumors and corresponding normal kidney tissues (**A, left panel**). High expression levels of GPNMB mRNA and protein in TFE3-fusion RCC-derived cell lines compared to negative protein expression in control papillary RCC-derived cell lines and control renal cell lines, Mann-Whitney test p = 0.004 vs. papillary RCC lines and p = 0.001 vs. all cell lines (**A, right panel**). Strong GPNMB immunohistochemical staining in TFE3-fusion RCC (**B**). Inhibition of cell viability by anti-GPNMB antibody-drug-conjugate CDX-011 (10ug/ml) in GPNMB-expressing TFE3-fusion cell lines vs. non-GPNMB expressing control cells (UOK140 – in lighter colors) (**C**). CDX-011 treatment of UOK124 xenografts caused a decrease in tumor volume (**D**), resulting in a highly significant decrease in growth rate measured as rate-based T/C metric (**E**). Animal survival probability was significantly extended by CDX-011 treatment (**F**)

In agreement with our previously published data [21], publicly available gene expression data from the Cancer Genome Atlas (TCGA) revealed that expression of *GPNMB*, a transcriptional target of TFE3, was significantly higher in TFE3-fusion RCC compared to clear cell RCC, papillary RCC or normal kidney [8, 35] (Supplementary Figure S7). *GPNMB* expression was elevated in TFE3-fusion RCC at the mRNA and protein level compared to very low expression in corresponding normal kidney tissues as confirmed by real-time PCR and western blot (Fig. 4A, left panel). GPNMB protein expression by IHC was high in the TFE3-fusion RCC tumor tissues that gave rise to the TFE3-fusion cell lines, but was largely negative in adjacent normal kidney parenchyma (Fig. 4B). Notably, two TFEB-fusion RCC samples from the TCGA dataset [8] also showed high GPNMB mRNA expression (Supplementary Figure S7), suggesting that this protein may be a potentially useful marker for differential diagnosis of MiT-fusion RCC. In addition, GPNMB, a cell surface protein, also serves as a potential therapeutic target, since a fully human antibody-drug conjugate (ADC) has been developed that targets GPNMB and is being evaluated for treatment of several cancers [19]. Here, we tested this ADC as a potential therapeutic option against MiT-RCC. A panel of patient derived RCC cell lines was evaluated for *GPNMB* gene and protein expression to determine if our TFE3-fusion RCC cell lines would prove to be valuable models for testing the GPNMB ADC. While GPNMB protein was virtually absent in cell lines derived from clear cell (shown in [21]) and papillary RCC, the protein was significantly elevated in TFE3-fusion RCC-derived cell lines (Fig. 4A, right panel; Mann-Whitney test p = 0.004). Flow cytometry analyses showed that GPNMB was localized to the cell surface and recognized by a commercial GPNMB antibody and by CR011, the antibody used to construct the ADC CDX-011 (Supplementary Figure S8A-C). CDX-011 is comprised of a GPNMB antibody linked to the cytotoxic drug monomethyl auristatin E (MMAE), a dolostatin 10 analog that blocks tubulin polymerization [36]. While the unconjugated tubulin inhibitor MMAE dramatically reduced cell viability of TFE3-fusion RCC cells and control cells at low nanomolar concentrations (Supplementary Figure S8D), the ADC showed significantly reduced cell viability only in TFE3-fusion RCC cells (Fig. 4C). To further verify that CDX-011 exerts a specific action on GPNMB-expressing cells, we performed an LDH release-based cytotoxicity assay and found that the ADC had a cytotoxic effect on GPNMB-positive UOK124 cells, but no effect on UOK140 GPNMB-negative control cells (Supplementary Figure S8E). In 3D spheroid cultures, CDX-011 affected volume, density and cell viability of TFE3-fusion RCC spheroids (UOK124) with minimal effect on the GPNMB-negative control cell line (Supplementary Figure S8F).

In vivo efficacy of the ADC was tested in UOK124-derived xenografts treated with four doses of CDX-011 (2.5 mg/kg, q4d, i.v.) or vehicle [37]. Treated mice demonstrated a significant decrease in tumor growth as compared to control mice (Fig. 4D,E). Mouse survival was significantly increased by CDX-011 treatment (Log-rank test p < 0.0001), without effect on animal weight(Fig. 4F; Supplementary Figure S8G).

### Combination treatments demonstrate synergistic therapeutic effects in TFE3-fusion RCC preclinical models

**Fig. 5:**
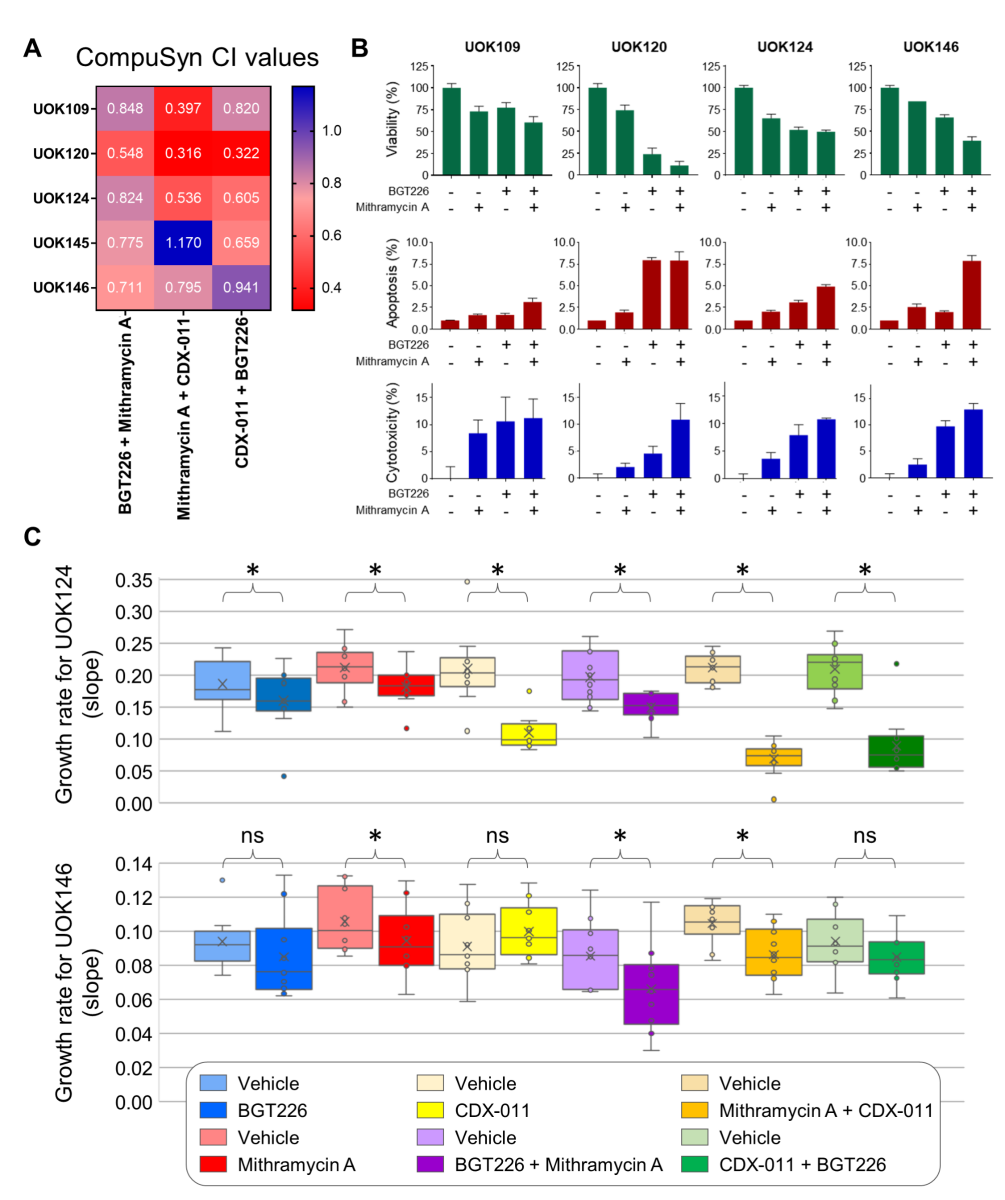
Drug combinations enhance efficacy of NVP-BGT226, Mithramycin A and CDX-011 against TFE3-fusion RCC. Combination viability studies were performed with TFE3-fusion RCC cell lines in 2D culture. Drug synergy was calculated with Compusyn and resulting CI values are plotted in a heatmap (**A**). Percent cell viability (**B, upper panel**), apoptosis signal (**B, middle panel**) and cytotoxicity (**B, lower panel**) were assessed all together by ApoToxGlo assay upon treatment of cells with 1µM Mithramycin (MTA) or 300nM NVP-BGT226 (BGT), singly or in combination. Results of in vivo mouse xenograft studies (**C**) with UOK124 and UOK146 cell lines are plotted as rate-based T/C metric for Mithramycin A, NVP-BGT226, CDX-011, all pairwise combinations of these drugs, and respective vehicle controls. Significantly different values (< 0.4) are indicated with *

Recent preclinical and clinical data suggest that drug combinations can benefit cancer patients. We combined the most promising drugs from our screening effort, Mithramycin A, NVP-BGT226 and the antibody-drug conjugate CDX-011 in vitro and in vivo to test if drug combinations could enhance therapeutic response against MiT-RCC. Cell culture experiments demonstrated that combining Mithramycin A with NVP-BGT226 led to a synergistic decrease in viability as compared to single agents. Similarly, Mithramycin A and NVP-BGT226 increased the drug efficacy of CDX-011 in vitro in most TFE3-fusion RCC cell lines tested (Fig. 5A,B; Supplementary Figure S9A). The combination of NVP-BGT226 with Mithramycin A exacerbated the inhibitory effect on mTOR and Akt (Supplementary Figure S9B). Concomitant with a synergistic effect on cell viability, combining the PI3K/mTOR inhibitor NVP-BGT226 with the RNA synthesis inhibitor Mithramycin A increased the cytotoxicity and induced apoptosis in TFE3-fusion RCC cell lines (Fig. 5B; Supplementary Figure S9C). Similarly, UOK124- and UOK146-derived xenograft studies showed a significant effect of combining Mithramycin A and NVP-BGT226 as compared to the respective vehicle controls or the single agents alone (Fig. 5C). While the anti-GPNMB ADC CDX-011 alone was more potent than NVP-BGT226 and Mithramycin A as single agents in the UOK124-derived xenografts, the combination of either of the two small molecule inhibitors with CDX-011 were synergistic in vivo. While CDX-011 alone had little effect in UOK146-derived xenografts and only showed some evidence of synergism in combination with Mithramycin A, the most effective combination in UOK146 was Mithramycin A and NVP-BGT226 (Supplementary Figure S9D-G; Supplementary Table S2). These data suggest that MiT-fusion RCC may benefit from a combination of drugs that would target different pathways to increase their efficacy, but that some variation in response could be expected.

## Discussion

MiT-RCC constitutes a subset of primarily sporadic RCC characterized by genomic translocations leading to tumor-driving fusion proteins involving members of the MiT family of transcription factors [4]. Although infrequently diagnosed in the adult population, MiT-RCCs represent a significant proportion of RCC in children and young adults. While these aggressive tumors have a propensity toward early metastasis to regional lymph nodes [4], currently no effective standard of care therapy is available. Therapeutic regimens developed to treat metastatic ccRCC, such as multi-kinase inhibitors and immune checkpoint inhibitors, have shown limited response in patients with metastatic MiT-RCC [11, 38, 39]. In an era of personalized medicine, the identification of a targeted therapeutic approach for the treatment of advanced MiT-RCC is needed. In the present study we have used a combination of an unbiased drug screening and biologically targeted approach.

The development of treatments for advanced rare tumor types always presents a challenge due to the scarcity of patients and, in this case, the severity of the disease and its rapid progression. For MiT-RCC patients, surgery remains the most effective treatment for localized disease since an effective systematic treatment for advanced, metastatic disease is still lacking. We have developed a series of TFE3 translocation RCC derived cell lines that represent different TFE3 fusions [2, 22, 23], which were used here in a high-throughput broad spectrum drug screen and to evaluate a GPNMB-targeted antibody-drug conjugate therapy.

The initial high-throughput drug screen and subsequent 2D and 3D spheroid-based validations highlighted the PI3K/mTOR inhibitor NVP-BGT226, the RNA synthesis inhibitor Mithramycin A, and the SRC inhibitor Dasatinib as promising therapies for MiT-RCC. On-target effects were confirmed for all three agents in the in vitro cell line models. Evaluation of these drugs in mouse xenografts derived from two different TFE3-RCC cell lines demonstrated growth suppression in both models with NVP-BGT226 and Mithramycin A, but growth suppression in only one model with Dasatinib. The efficacy of the PI3K/mTOR inhibitor NVP-BGT226 correlates well with recent studies that proposed Akt/mTOR inhibition as a promising therapeutic target for TFE3-fusion RCC [26, 40]. In line with these previous studies, NVP-BGT226 decreased the S-phase of the cell cycle while not significantly inducing apoptosis in TFE3-RCC cell lines [26, 40]. These studies support the potential use of PI3K/mTOR inhibitors to treat MiT-RCC, most likely as a component of combination therapy.

This study confirms growth suppression by Mithramycin A in MiT-RCC cells. A selective growth-inhibitory effect of Mithramycin A has been observed in *FLCN*-deficient tumor cells as well [41],which are characterized by activated wild-type TFE3 [20], suggesting that TFE3-driven tumor cells may be specifically sensitive to this drug. Mithramycin A is an anti-tumoral antibiotic that binds to GC-rich regions of DNA and inhibits RNA synthesis by blocking the DNA binding capacity of transcription factors, such as Specificity Protein 1 (SP1) [42] or E2F [43], thereby leading to reduced cell proliferation and survival. A known transcriptional target of SP1, *BIRC5* (survivin) [44], was upregulated in TFE3-RCC. This study confirms the expected decrease in SP1 transcriptional activity including *BIRC5* expression in response to Mithramycin A treatment. The treated TFE3-RCC cells demonstrated a G2/M phase block in the cell cycle and increased apoptosis as was previously observed in ovarian carcinoma cells in response to a Mithramycin A analog [43]. Mithramycin A has shown relatively high toxicity in patients at therapeutic doses and Mithramycin A analogs with better toxicity profiles, such as EC-8042 (EntreChem SL), have been developed [34]. Although TFE3-RCC cell lines were less sensitive to EC-8042 in comparison to Mithramycin A, the improved toxicity profile suggests its potential use as a component of combination therapy without excessive toxicity. Additionally, these results support further investigation of additional mithralogs as potential therapeutic agents for MiT-RCC.

The unbiased high-throughput drug screen identified pharmacological targets for TFE3-RCC from an array of known anti-tumor agents but was not directed against specific dysregulated pathways in the tumor. Improved understanding of MiT-RCC tumor biology may elucidate specific and untested therapeutic targets or vulnerabilities. In this study the observed tumor specific expression of the cell surface marker GPNMB led to the preclinical evaluation of Glembatumumab vedotin (CDX-011), a fully human antibody-drug conjugate (ADC) that targets GPNMB and delivers a cytotoxic dolostatin-like tubulin inhibitor, Monomethyl auristatin E (MMAE) [36, 37]. CDX-011 has been previously shown to be safe for clinical use and demonstrates some pharmacologic effects against breast cancer, melanoma, and osteosarcoma [45–50]. CDX-011 induced growth inhibition of TFE3-RCC both in vitro and in vivo, while having no effect in vitro on a cell line derived from a clear cell RCC. In support of GPNMB as a therapeutic target in cancer, a previous study of ASPSCR1-TFE3 fusion-driven alveolar soft part sarcoma also demonstrated increased GPNMB expression, and *Gpnmb* silencing in a mouse model of this disease inhibited cell migration, suggesting a role in metastasis [51].

While NVP-BGT226, Mithramycin A and CDX-011 induced growth inhibition as single therapies in this study, dual agent combinations demonstrated improved responses compared to single agents in both TFE3-RCC xenograft models studied. Recent advances in treating RCC have placed a substantial emphasis on combination therapies, even as a first line therapy [52]. Combination therapies allow for different tumorigenic pathways to be targeted simultaneously with an increased likelihood of therapeutic response and decreased opportunity for cancer cells to develop resistance. Advanced MiT-RCC is very aggressive and may benefit greatly from combination therapy if the side effects can be limited, for example, by using precision therapies such as CDX-011.

A limitation of this study is the use of cell line models in evaluating potential therapies. These cell lines models are artificially cultured, may have acquired additional genetic alterations, and may not accurately represent the wide variety of tumors classified as MiT-RCC. For MiT-RCC, this is particularly notable as the tumors are driven by gene fusions involving several members of the MiTF gene family fused to a diverse number of other partner genes. To partially counteract some of these disadvantages, this study utilized multiple cell lines that represented three different TFE3 fusion partners, NONO, PRCC, and SFPQ. Several other fusion combinations have been identified and it is possible that these other gene fusions may not respond in the same way to the potential therapies described in this study. Even the subset of TFE3-RCC tumors show high clinical and histological variability, which may in part correlate with the TFE3 fusion partner. Successful drug design and treatment strategies will therefore require a personalized medicine approach involving the molecular identification of the MiT-RCC subtype and potentially additional biomarkers (e.g., GPNMB expression levels) for each patient. Further investigations using patient derived xenografts (PDXs), potentially in humanized mice, may provide a more comprehensive representation of MiT-RCC in preclinical studies.

## Conclusions

In this study multiple TFE3-RCC tumor-derived cell lines representing different TFE3 fusions were utilized to generate in vitro and in vivo preclinical data supporting the efficacy of PI3K/mTOR inhibitor NVP-BGT226, transcription inhibitor Mithramycin A, and GPNMB-targeted antibody-drug conjugate CDX-011 as potential therapeutic options for treating advanced MiT-RCC. The results of this study should provide the basis for designing future clinical trials for patients with MiT-driven RCC.

## Electronic supplementary material

Below is the link to the electronic supplementary material.

Supplementary Material 1: Figures S1–S9

Supplementary Material 2: Table S1

Supplementary Material 3: Table S2

Supplementary Material 4: Supplemental Methods

## Data Availability

All data generated or analyzed during this study are included in this published article and in its supplementary materials. Full high-throughput drug screening data are available upon request.
