# Supplementary material for: High-throughput and targeted drug screens identify pharmacological candidates against MiT-translocation renal cell carcinoma
Source: J Exp Clin Cancer Res. 2023 Apr 25;42:99. doi: 10.1186/s13046-023-02667-4 (PMC10127337; doi:10.1186/s13046-023-02667-4)
Supplement: Supplementary file 1 — Supplementary Material 1: Figures S1–S9 [file 13046_2023_2667_MOESM1_ESM.docx]

­­
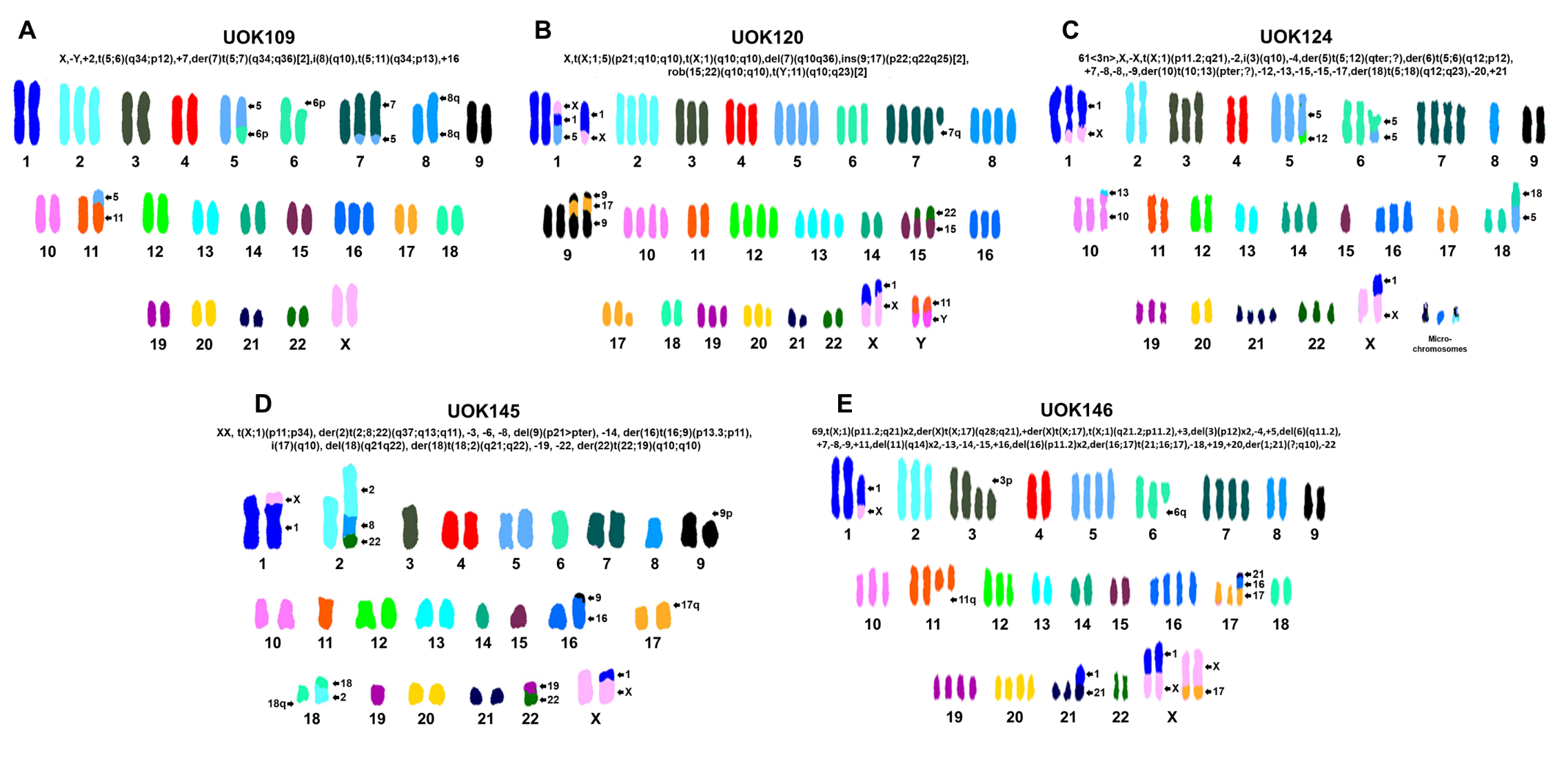


**Supplementary Figure S1: Spectral karyotype (SKY) of TFE3-fusion RCC cell lines**

SKY analysis of UOK109, UOK120, UOK124, UOK145, UOK146 (A-E, respectively) demonstrated chromosomal level alterations, including translocations involving chromosome X, leading to TFE3 gene fusions.


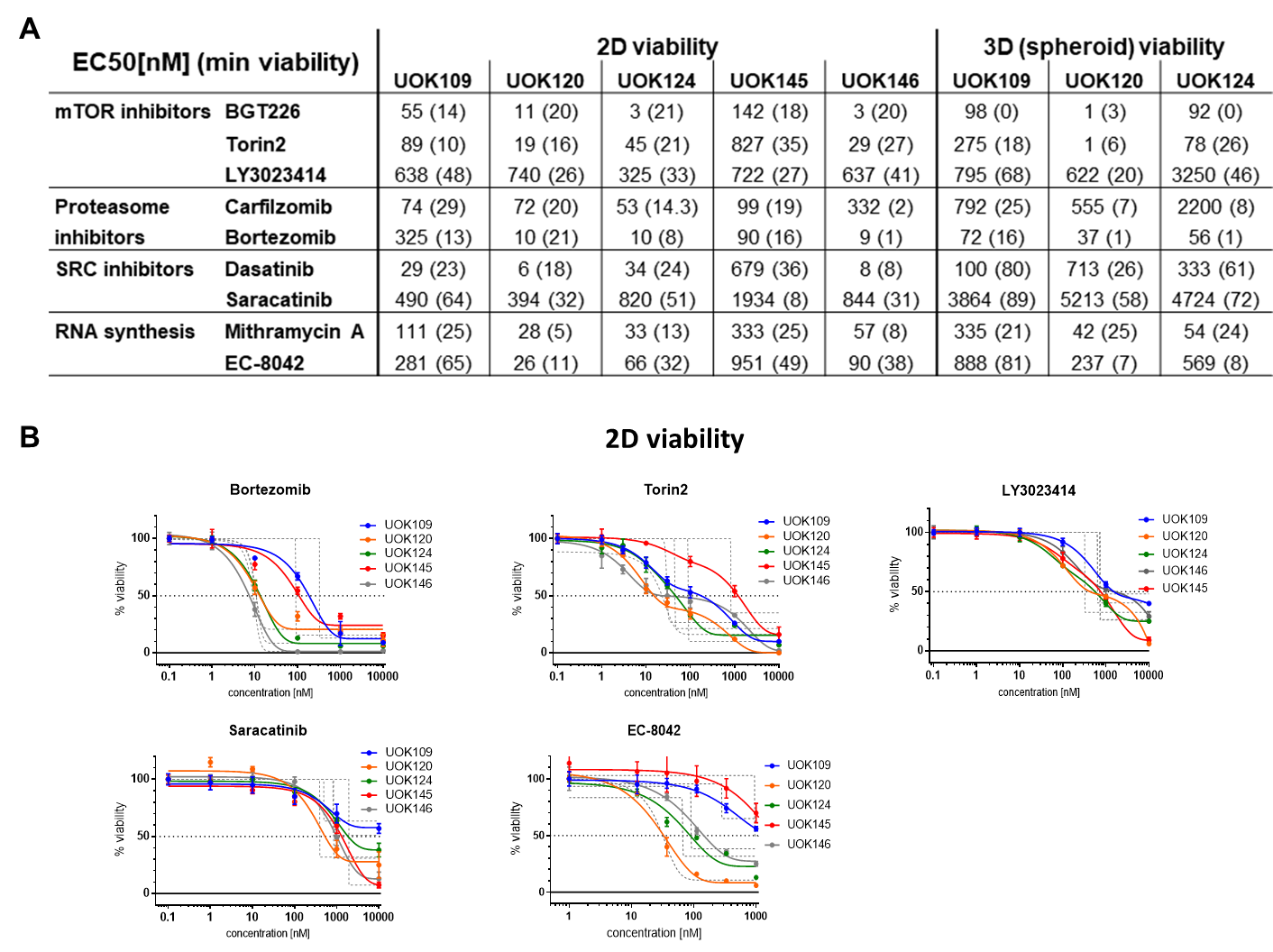

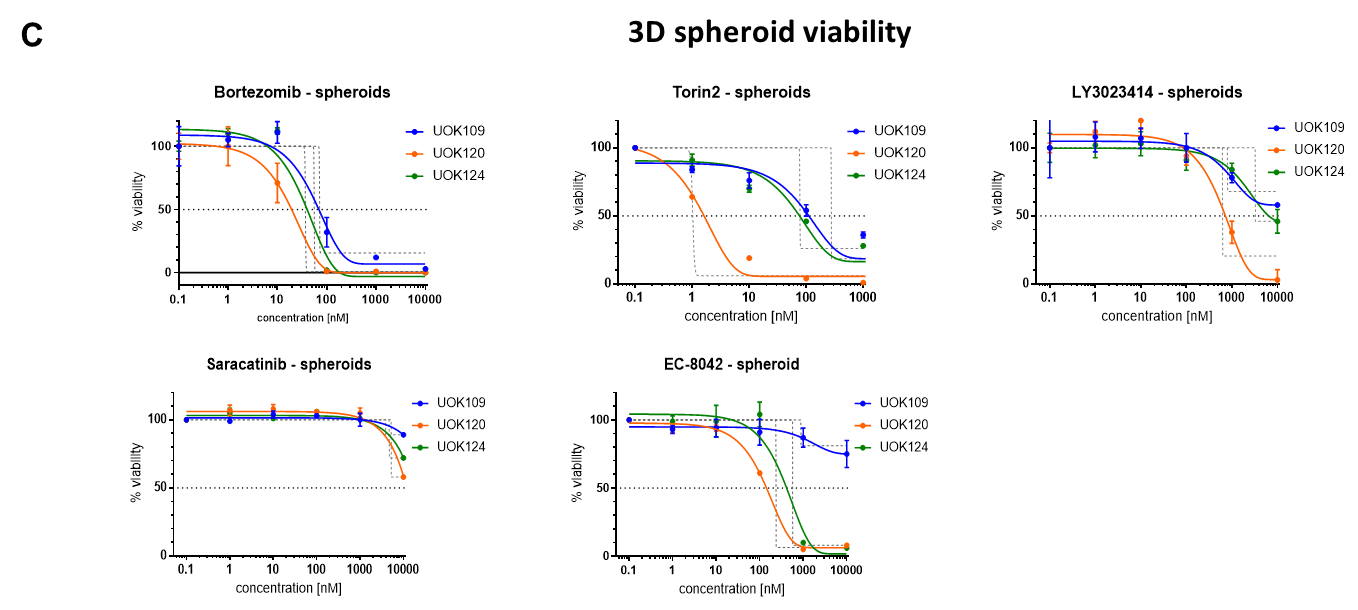


**Supplementary Figure S2: 2D and 3D spheroid cell viability analyses**

A table showing the EC50(nM) and minimum viably cells percentage at maximum dose for candidate drugs derived from in-house 2D and 3D confirmations (**A**). 2D cell viability curves for 5 selected drug candidates in 5 TFE3-fusion RCC cell lines (UOK109, UOK120, UOK124, UOK145, UOK146) (**B**). EC50 log agonist vs. response curves are shown in gray dotted lines. 3D spheroid viability curves for 5 selected drug candidates in three spheroid-producing TFE3-fusion RCC cell lines (UOK109, UOK120, UOK124) (**C**). EC50 log agonist vs. response curves are shown in gray dotted lines.


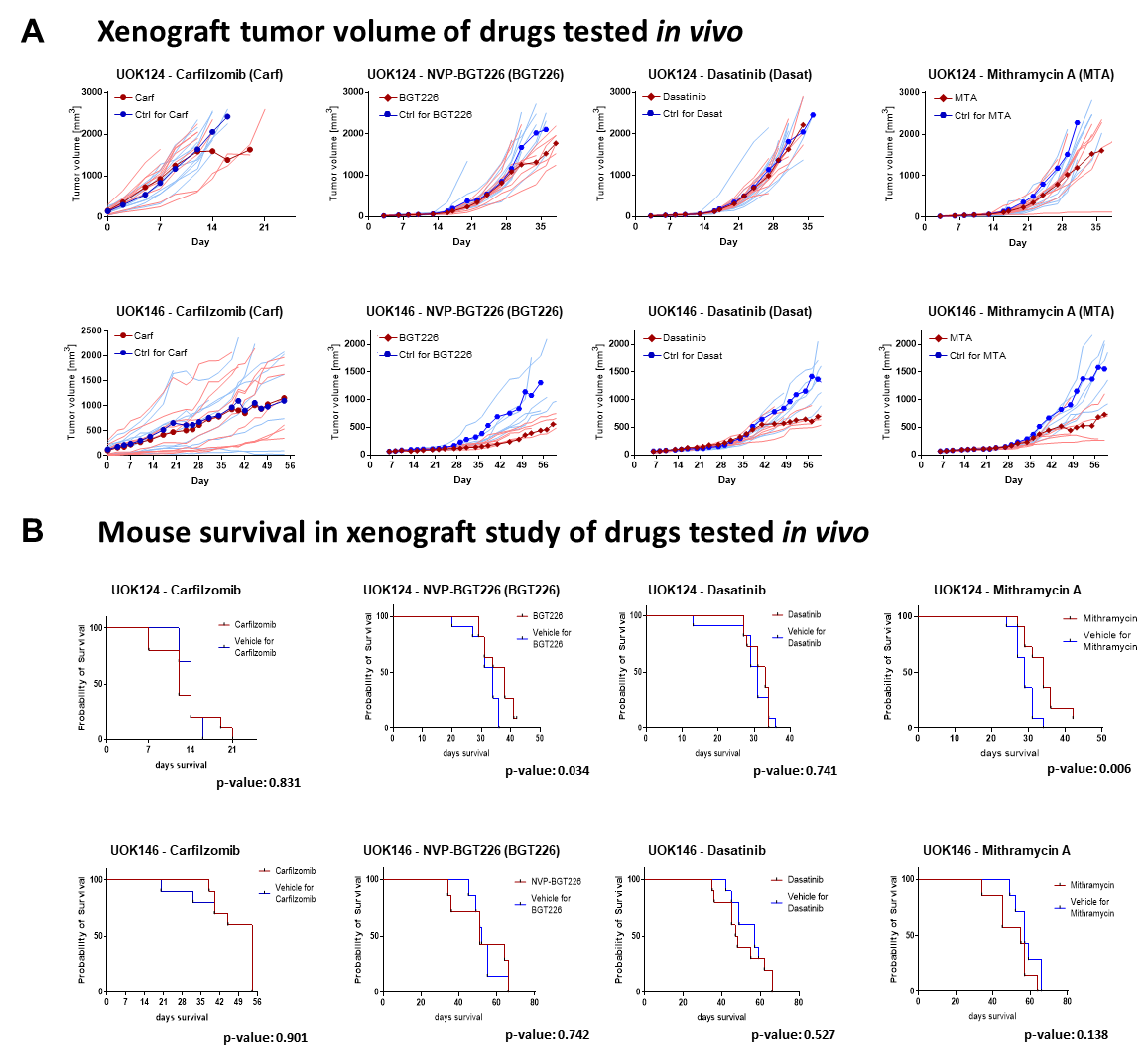

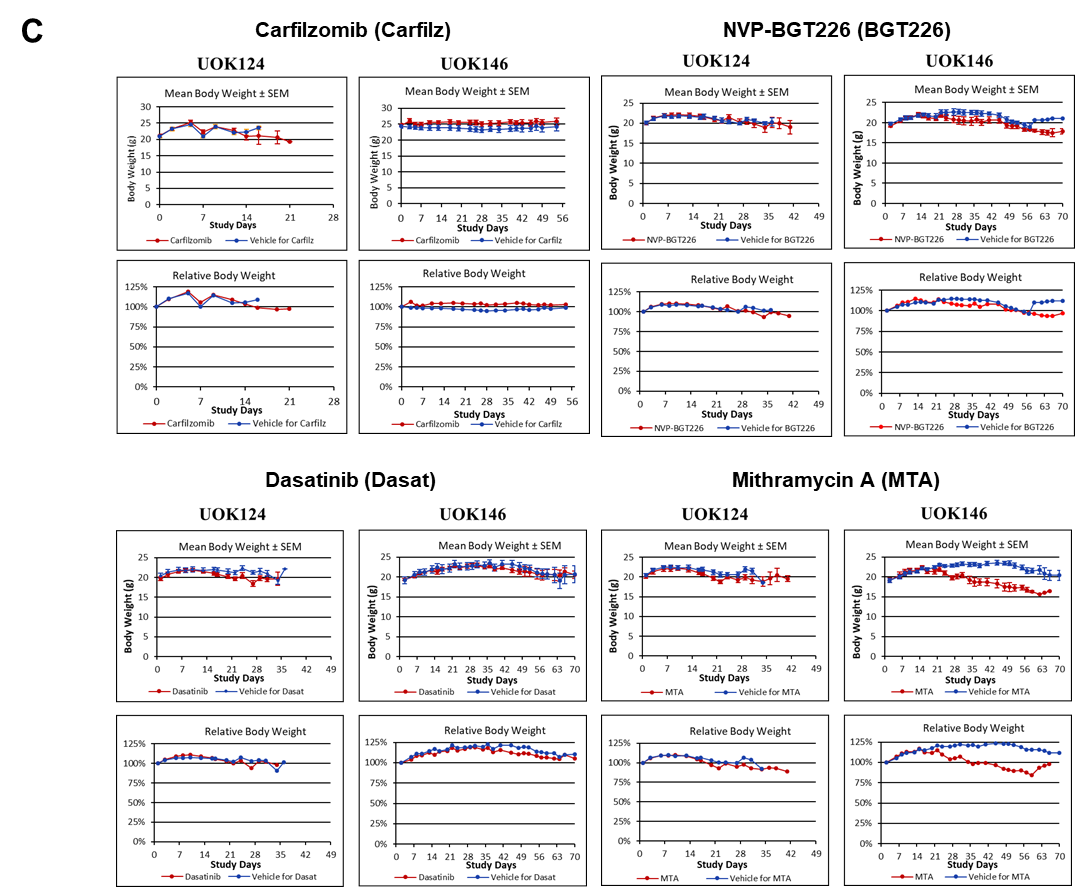


**Supplementary Figure S3: In vivo screening result of 4 drugs in UOK124 and UOK146 xenograft models.**

Tumor growth curves for drug-treated (red) and vehicle treated (blue) xenografts are shown for UOK124 and UOK146 models (**A**). Bold line represents average tumor volume, while fine lines represent single tumors. Mouse survival calculated as Log-rank test is shown for 2 cell line models treated with 4 drugs (**B**). Animal weight measurements throughout the xenograft studies are shown as absolute and relative body weight (**C**). This measure was used as one parameter to monitor drug toxicity effects.


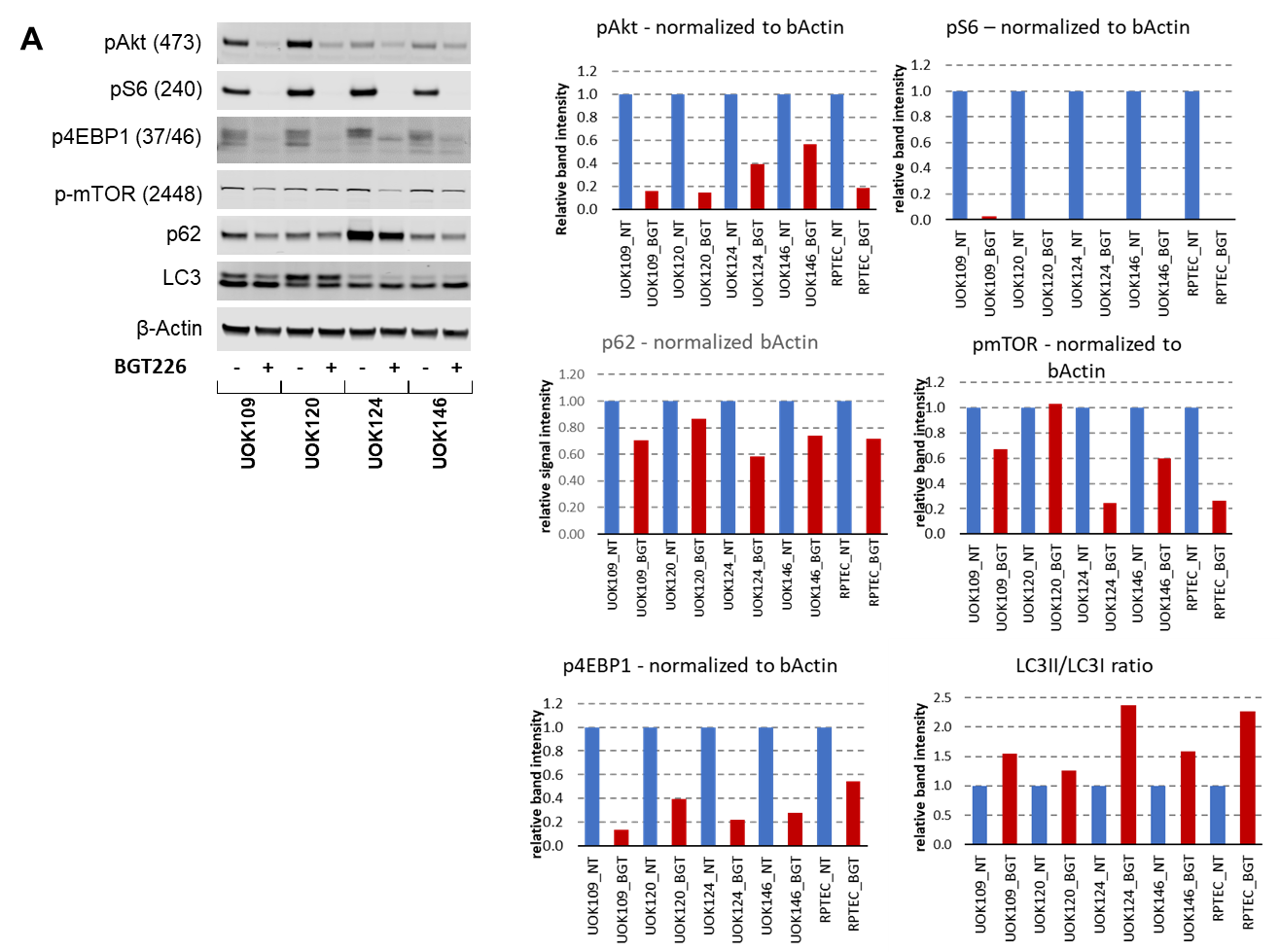


**
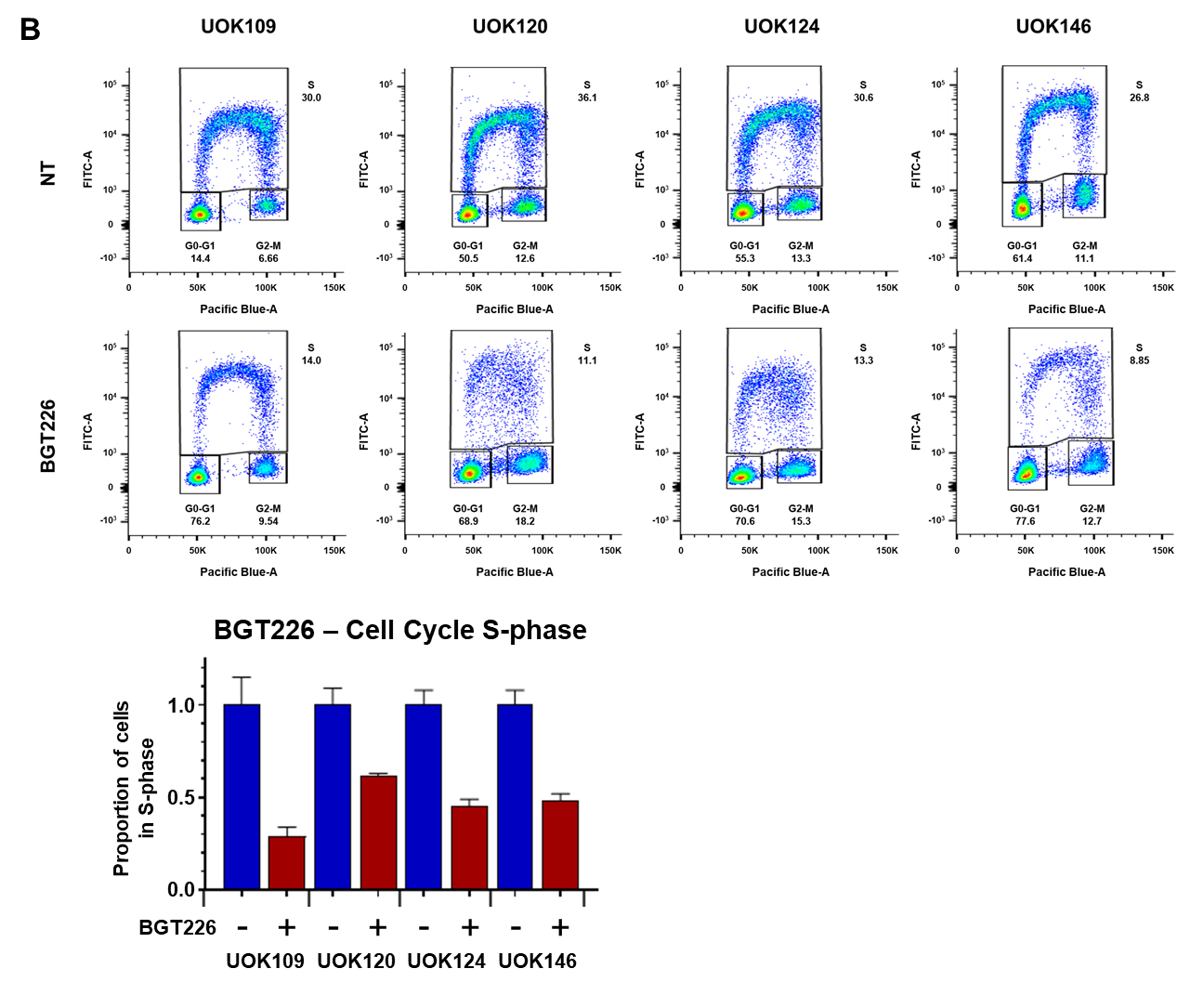
**
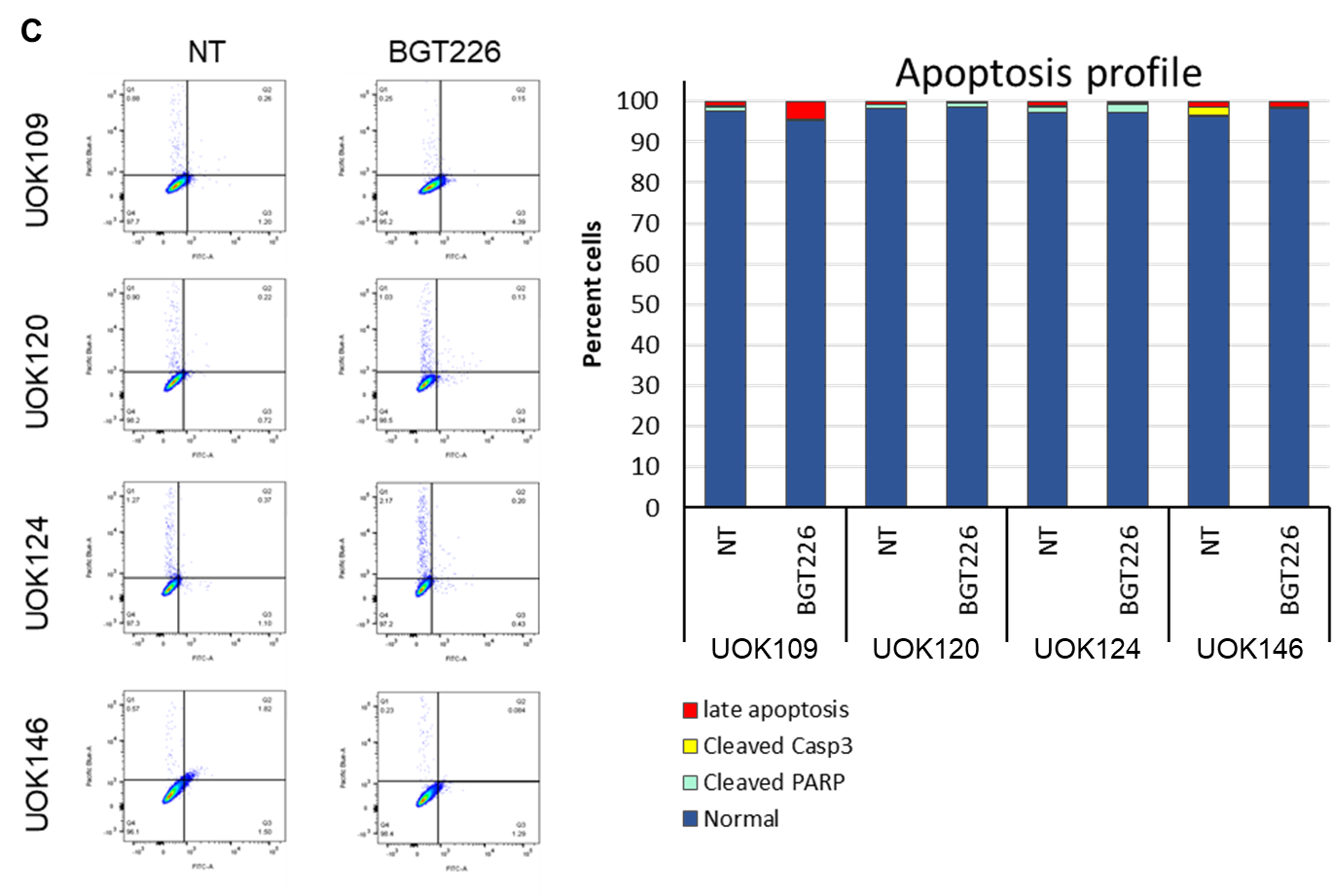
**Supplementary Figure S4: Drug mechanism of PI3K/mTOR inhibitor NVP-BGT226.**

Quantification of western blot bands as shown in Figure 4A (**A**). Dot plots and comparison of cell cycle S-phase (**B**) and apoptosis (**C**) upon 24-hour treatment with NVP-BGT226 are shown for UOK109, UOK120, UOK124 and UOK146 cells.


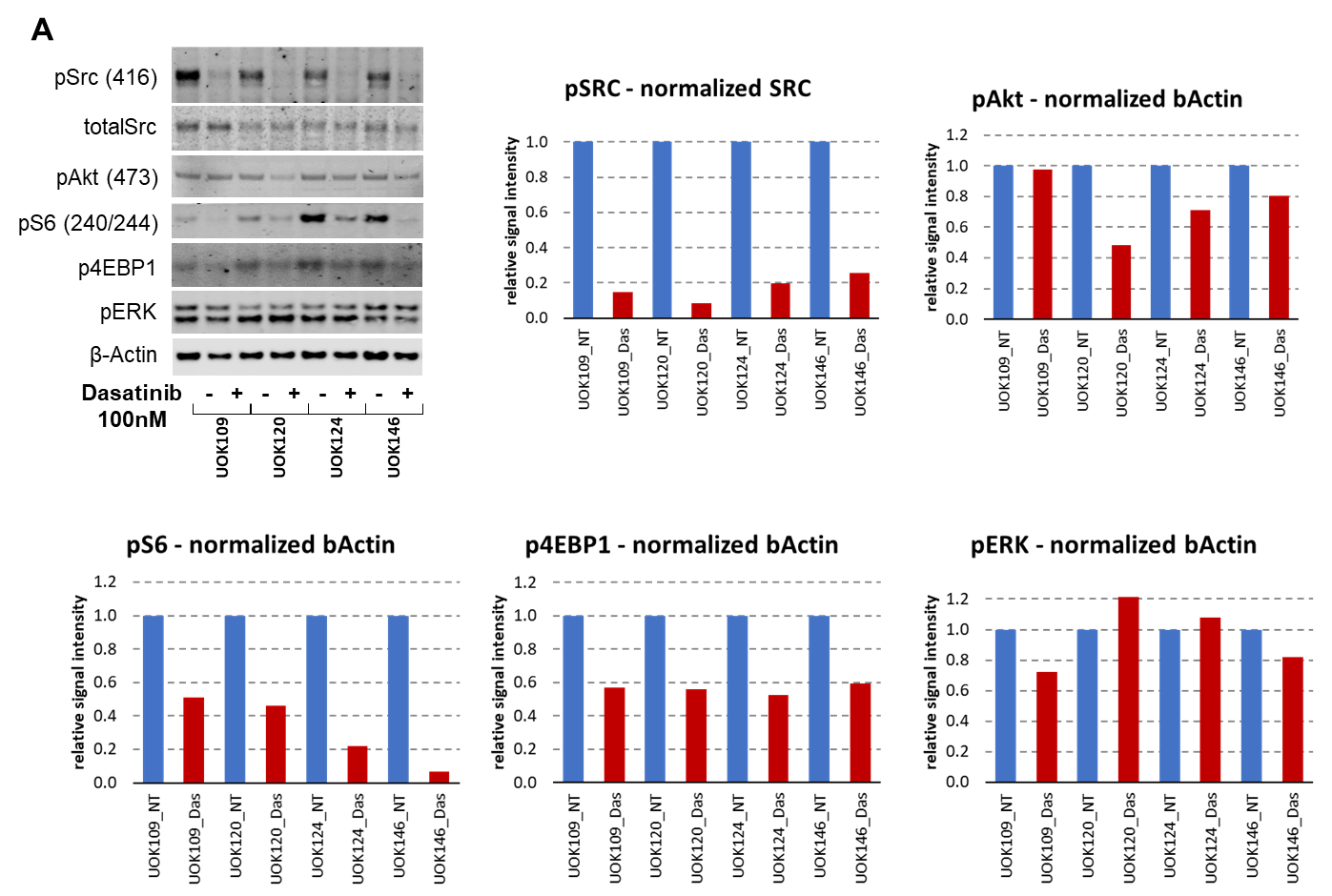

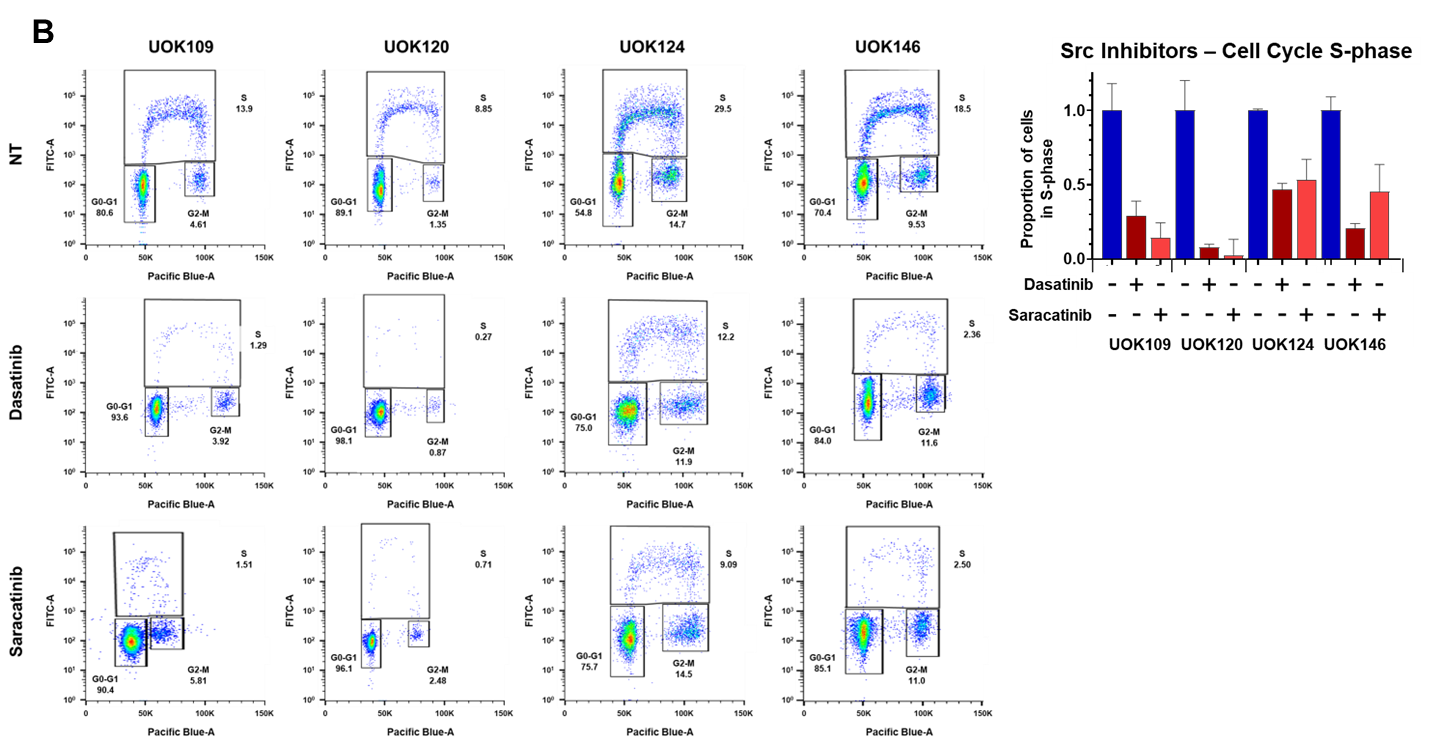


**Supplementary Figure S5: Drug mechanism of Src inhibitors Dasatinib and Saracatinib.**

Quantification of western blot bands as shown in Figure 4C (**A**). Dot plots and comparison of cell cycle S-phase of TFE3-fusion RCC cell lines untreated (Ctrl) or treated with Dasatinib and Saracatinib (**B**).


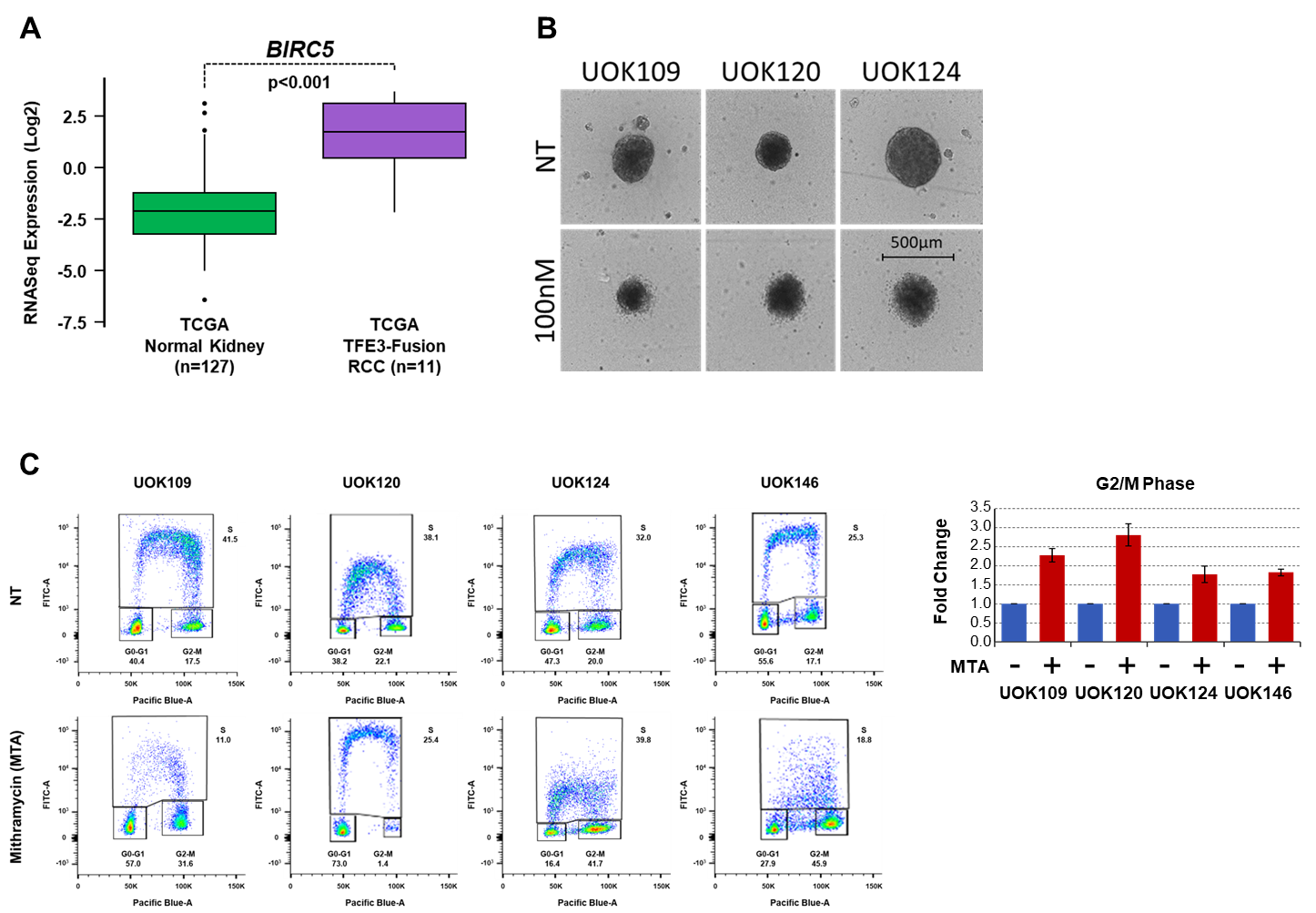

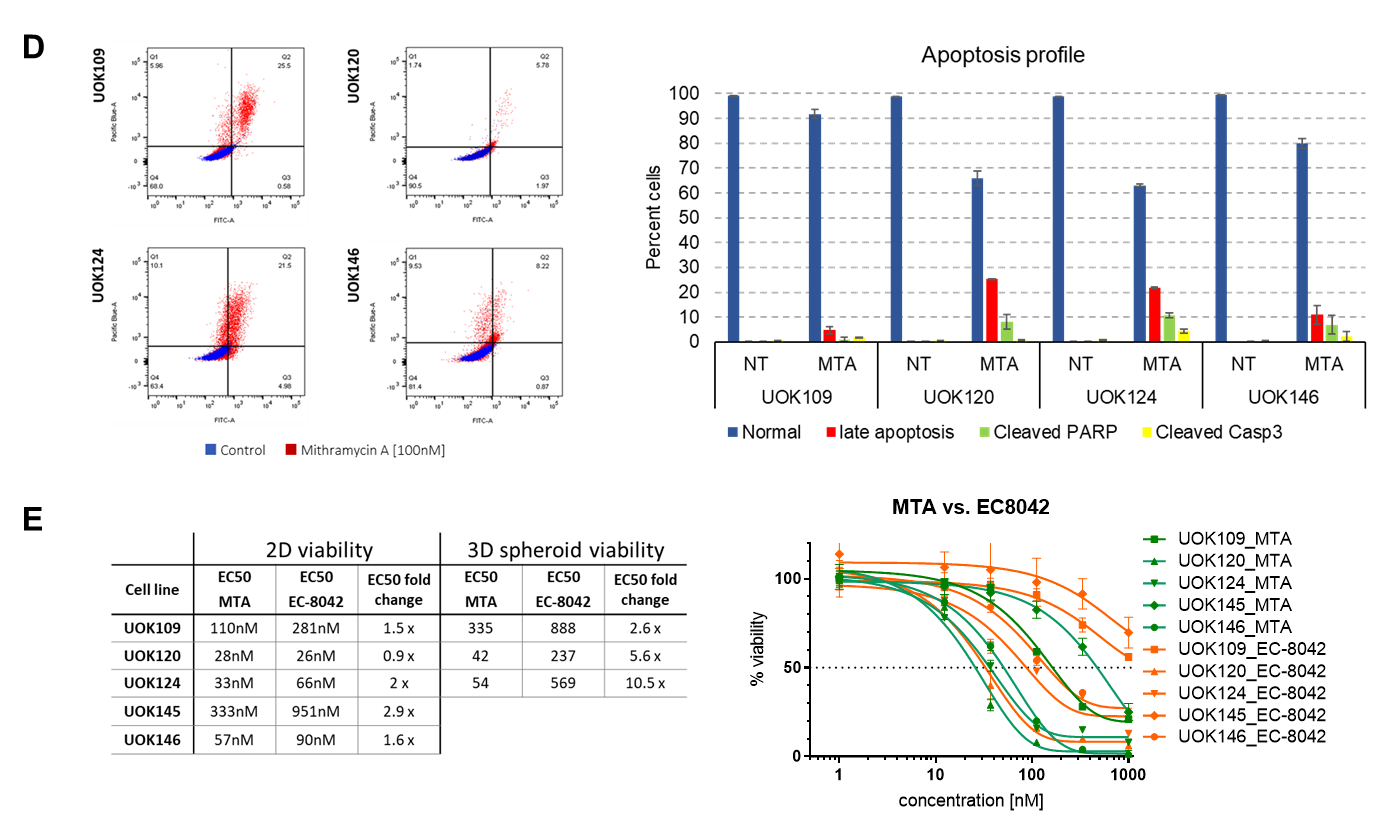


**Supplementary Figure S6: Drug mechanism of RNA synthesis inhibitor Mithramycin A.**

TCGA gene expression data for *BIRC5* in 11 TFE3-fusion RCC compared to normal kidney parenchyma tissues (**A**). Images of TFE3‐fusion cell line spheroids untreated (NT) and treated with 100nM Mithramycin for 5 days (**B**). Treated spheroids are significantly smaller and show “fuzzy” edges. Cell viability measurement confirmed decreased viability of treated spheroids. Dot plots and summary breakdown of cell cycle analysis (**C**) and apoptosis (**D**) of TFE3-fusion RCC cell lines untreated or treated with 100nM Mithramycin (MTA)**.** Viability comparison between Mithramycin and EC-8042, showing an up to 3‐fold lower potency of EC-8042 as compared to Mithramycin in 2D in vitro models, while displaying a ~3‐10‐fold lower potency in 3D spheroid models (**E**).

**Supplementary Figure S7: GPNMB is upregulated in MiT-RCC.**

*GPNMB* expression in RCC samples and normal kidney parenchyma from the TCGA project.


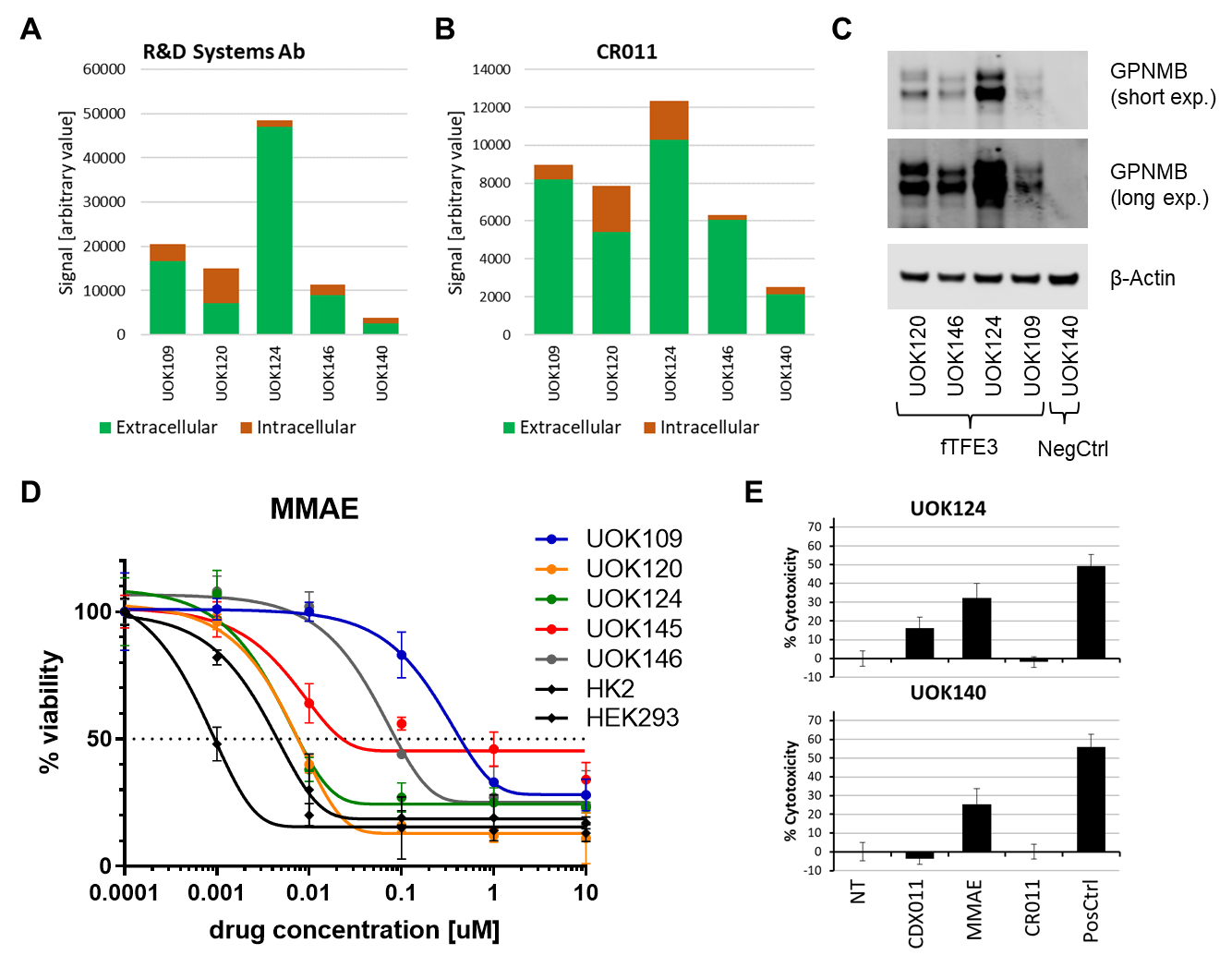


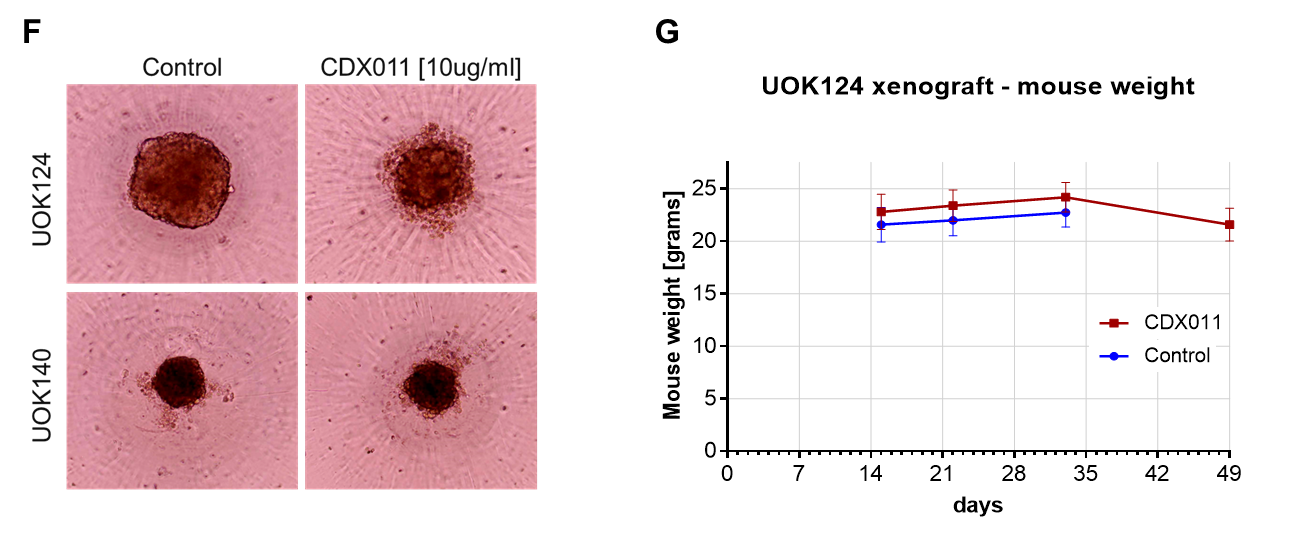


**Supplementary Figure S8: GPNMB as therapeutic target against MiT-RCC.**

Flow cytometry on permeabilized and non-permeabilized cells was applied to detect the expression of GPNMB on the cell surface of TFE3-fusion RCC cell lines and a GPNMB-negative control cell line (UOK140) with a commercial antibody against GPNMB (R&D systems #AF2550) (**A**) and the fully humanized antibody CR011 used to construct the antibody-drug conjugate CDX-011 (**B**). Western blot confirming positive and negative expression of GPNMB in cell lines used for flow cytometry experiment (**C**). Viability curves of TFE3-fusion RCC cell lines and commercially available normal kidney cell lines (HK2, HEK293) treated with increasing doses of dolostatin 10 analog monomethyl auristatin E (MMAE), the drug load conjugated to the antibody in the ADC CDX-011 against GPNMB (**D**). LDH release-based cytotoxicity assay was performed in UOK124 TFE3-RCC cells and UOK140 control cells to assess % cytotoxicity upon treatment with MMAE, fully humanized antibody CR011 and the antibody-drug conjugate CDX-011. Multi protein inhibitor LY294002 (100 μM) was used as a positive control. (**E**). Images of UOK124 and UOK140 (control) spheroids untreated and treated with 10ug/ml CDX-011 for 5 days, showing significant effect on spheroid size and shape of GPNMB-positive UOK124 cells (**F**), while barely affecting GPNMB-negative UOK140 cell spheroids. Mouse weight during UOK124 xenograft study of CDX-011 treated (red) and vehicle-treated (blue) animals (**G**).


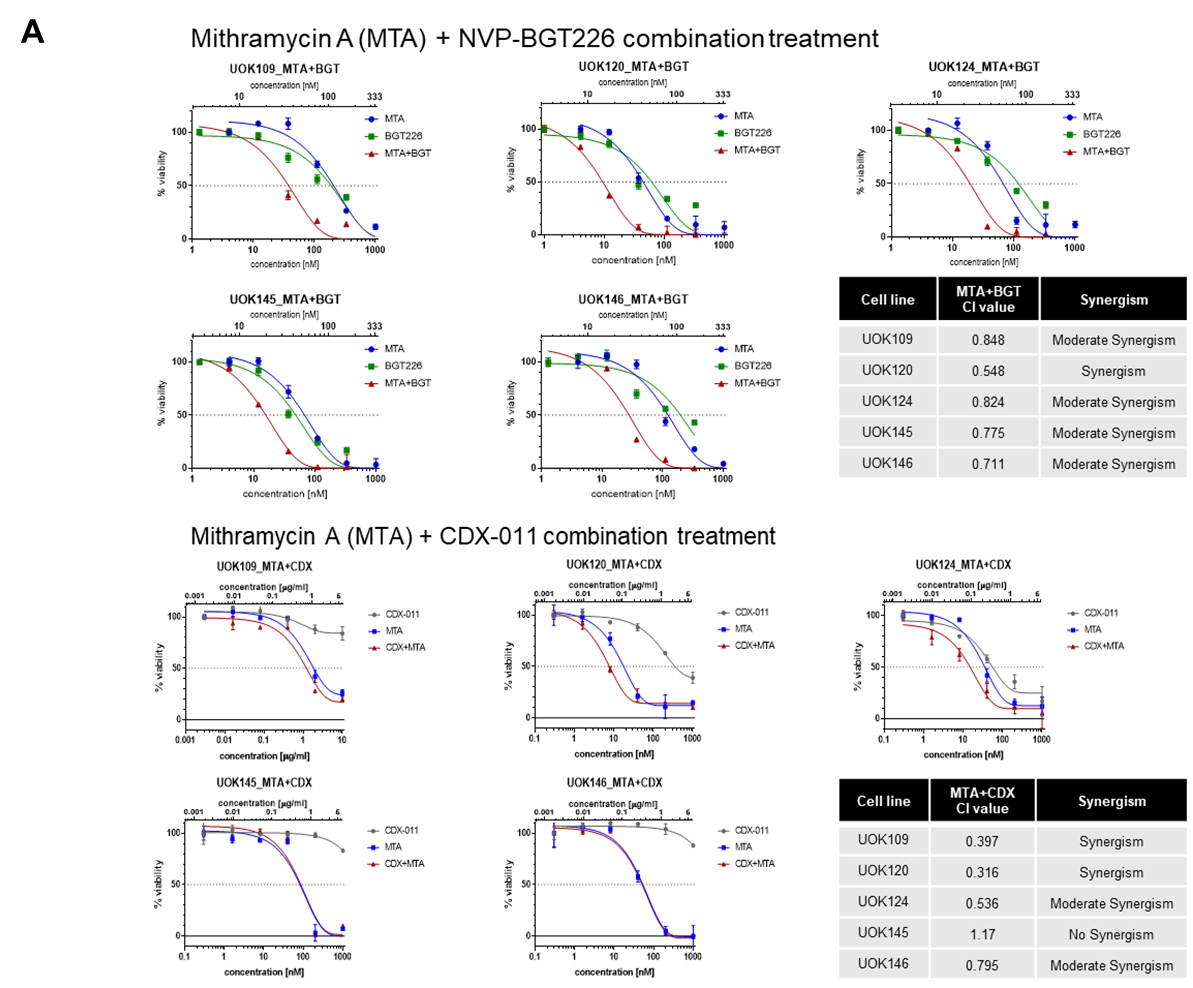


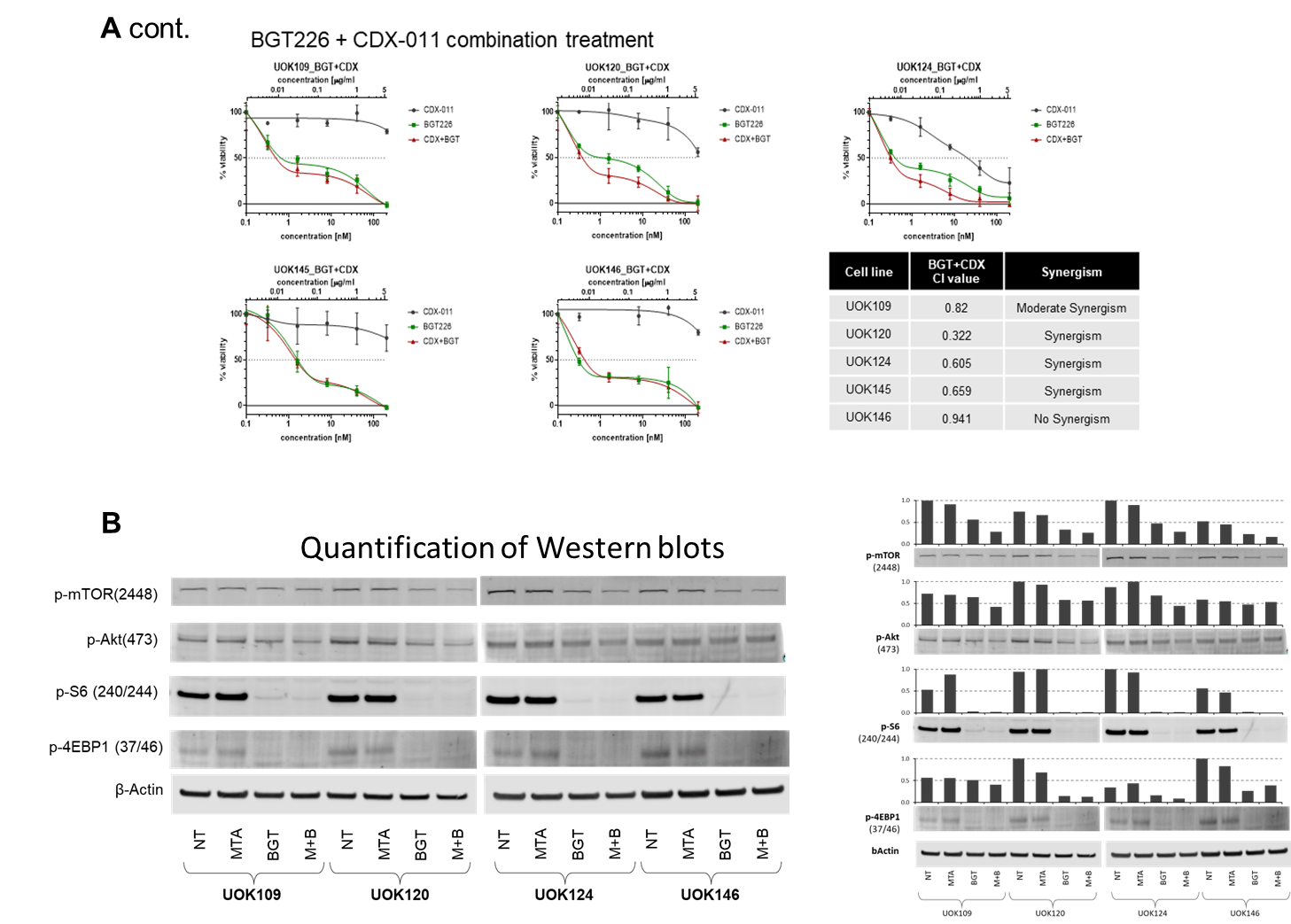

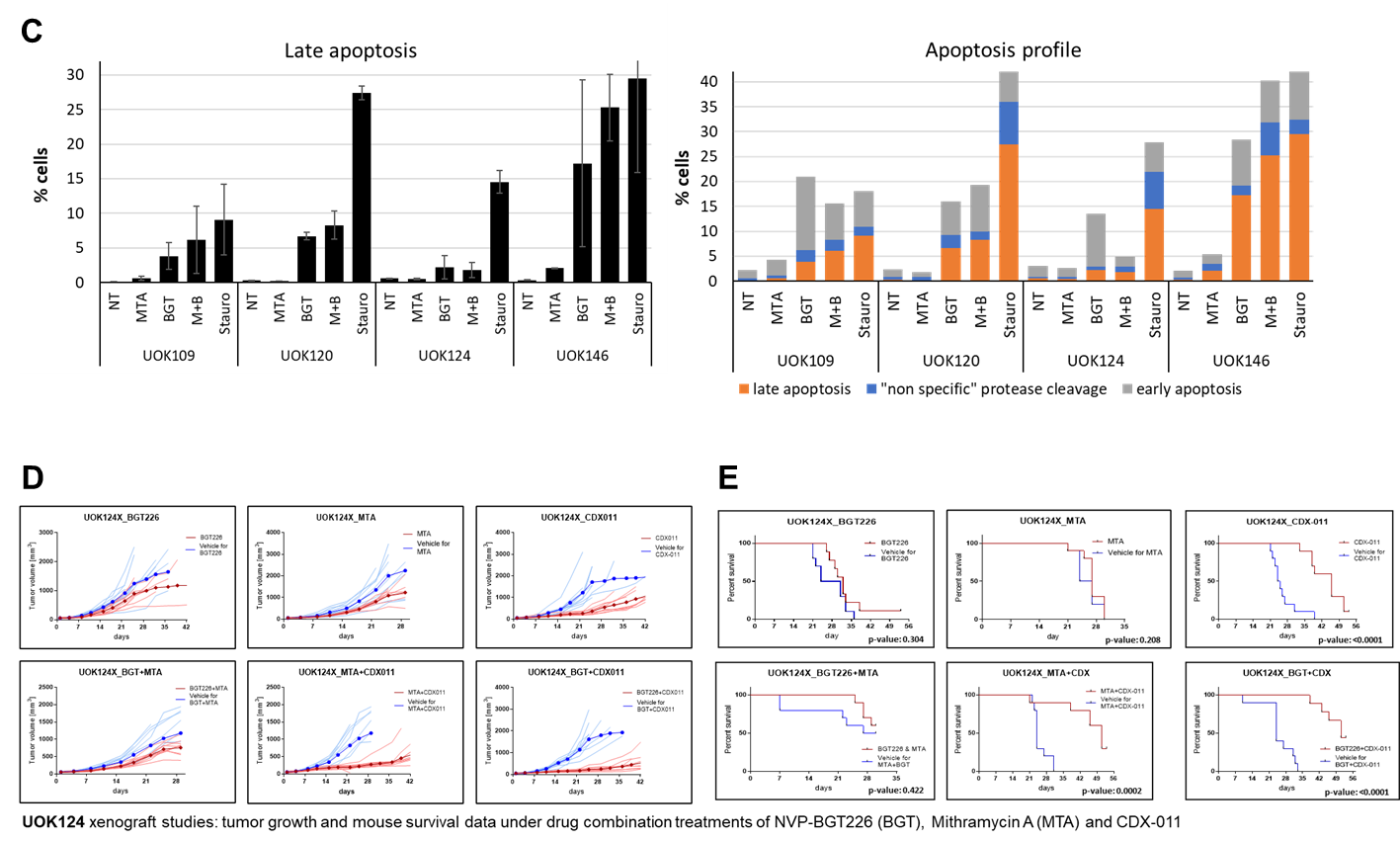

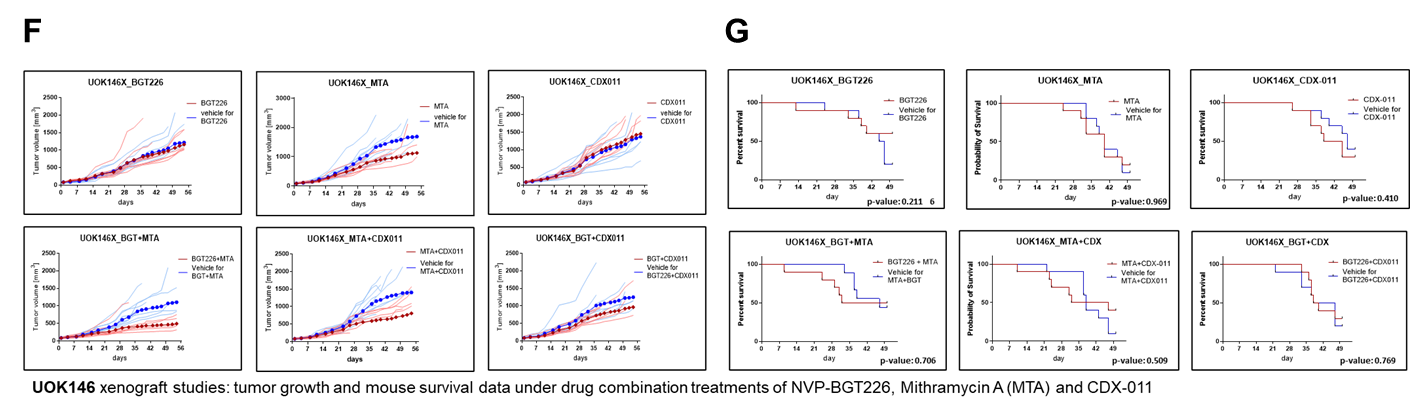


**Supplementary Figure S9: Drug combinations increase potency against TFE3-fusion RCC.**

Cell viability graphs are shown for 5 TFE3-fusion RCC cell lines subjected to combination treatments with NVP-BGT226, Mithramycin A and CDX-011 (**A**). CompuSyn CI values are shown for each drug combination and cell line. Western blot experiments of cell lines treated with Mithramycin A (MTA), NVP-BGT226 (BGT) or in combination (M+B) and quantification of western blot bands are presented (**B**). Percent of cells demonstrating late apoptosis signals and overall apoptosis profiles upon drug combination treatment with Mithramycin A (MTA), NVP-BGT226 (BGT), and in combination, as measured by flow cytometry with cleaved caspase 3 and cleaved PARP antbodies (**C**). Mouse xenograft studies were performed on UOK124 and UOK146 models with NVP-BGT226, Mithramycin A and CDX-011 and all respective two-way combinations. Tumor growth (**D, F**) and animal survival (**E, G**) calculated as Log-rank test are shown for UOK124 and UOK146 cell line models, respectively. Bold lines represent average tumor volume, while fine lines represent single tumors of drug treated (red) and vehicle treated (blue) animals.
