# Supplementary material for: High-throughput and targeted drug screens identify pharmacological candidates against MiT-translocation renal cell carcinoma
Source: J Exp Clin Cancer Res. 2023 Apr 25;42:99. doi: 10.1186/s13046-023-02667-4 (PMC10127337; doi:10.1186/s13046-023-02667-4)
Supplement: Supplementary file 3 — Supplementary Material 3: Table S2 [file 13046_2023_2667_MOESM3_ESM.docx]

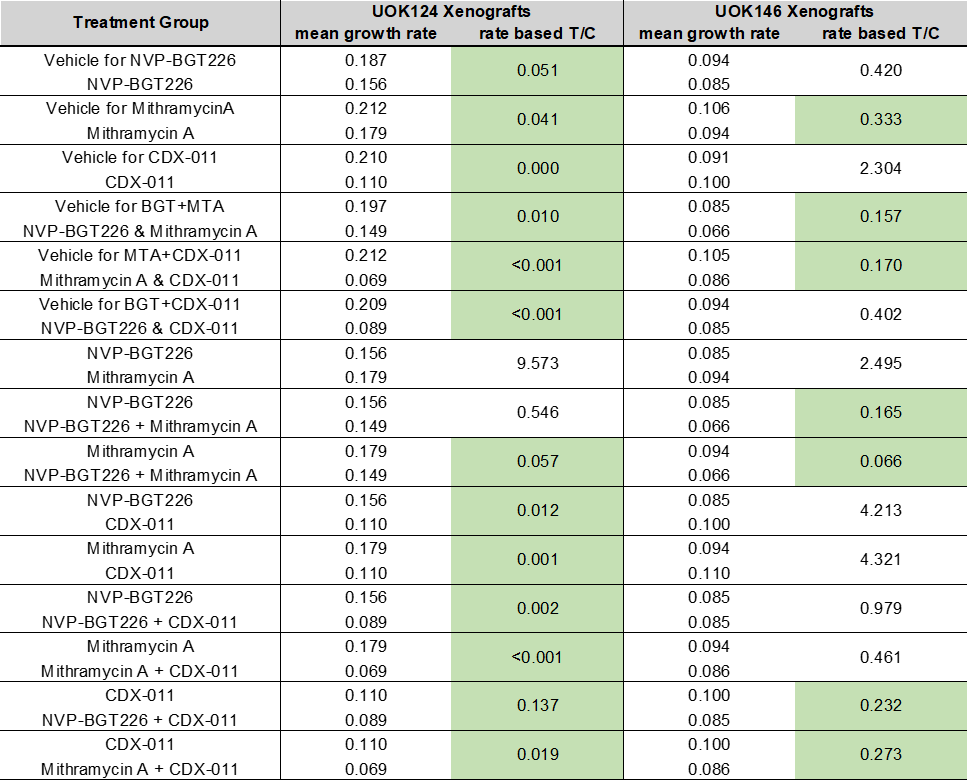


**Supplementary Table S2: Xenograft growth rate comparison in combination drug study.**

Comparison of xenograft growth rates under combination treatments with NVP-BGT226, Mithramycin A and CDX‐011 and respective vehicle controls. Growth rates were calculated by fitting each tumor’s growth to an exponential model according to (Hather, Liu et al. 2014) and rate-based T/C values were calculated for each pair as 10^(𝜇𝑇−𝜇𝑐)^ *  (number of study days), where 𝜇𝑇 is the mean growth rate of the treatment group and 𝜇𝑐 is the mean growth rate of the control group.
