# Supplementary material for: High-throughput and targeted drug screens identify pharmacological candidates against MiT-translocation renal cell carcinoma
Source: J Exp Clin Cancer Res. 2023 Apr 25;42:99. doi: 10.1186/s13046-023-02667-4 (PMC10127337; doi:10.1186/s13046-023-02667-4)
Supplement: Supplementary file 4 — Supplementary Material 4: Supplemental Methods [file 13046_2023_2667_MOESM4_ESM.docx]

# Supplementary Methods

**Quantitative High Throughput Screening**

In collaboration with the National Center for Advancing Translational Sciences (NCATS), a quantitative high throughput screening (qHTS) was conducted on three TFE3-fusion cell lines bearing three different fusion partners (UOK109, UOK124, UOK145). Briefly, 500 cells in complete growth media were plated into white, solid-bottom, 1536-well tissue culture treated plates (Grenier Cat. 789173-F) using a Multidrop Combi cassette dispenser. 23nl of 1912 pharmacologically defined small molecule compounds of the Mechanism Interrogation PlatE (MIPE) version 4.0 (1) were added to each plate using a Pintool Dispenser (Wako). Plates were covered with stainless steel gasketed lids and incubated at standard cell culture conditions. Viability was assessed by adding 3uL of CellTilter-Glo assay reagent (Promega) 48 hours post-treatment and plates were incubated for 15 minutes at room temperature. Luminescence was measured on a ViewLux plate reader (PerkinElmer). Post-screen data processing used DMSO controls as 100% viability and empty wells as 0% viability and dose response curve fitting was done for all 11 concentrations for each compound. Z-transformed area under the curve values were calculated for each compound in each cell line and hits were classified as any compound with a Z-AUC less than -0.8 in all 3 cell lines (2).

**SP1 Transcription factor reporter Assay**

SP1 reporter assay was performed with Cignal Luciferase Reporter Assay Kit (Qiagen) following manufacturer’s instructions. In brief, 5x10^3^ cells were seeded on 96-well plates, incubated 24 hours, transfected with 75ng plasmids (reporter, positive and negative control) and 0.2μl Lipofectamine 2000 (Thermo Fisher Scientific) in 100μl culture medium. 24 hours post transfection, cells were treated with 100nM Mithramycin and luciferase signal was detected after additional 24 hours by Dual-Glo® Luciferase Assay System (Promega) according to manufacturer’s recommendations.

**Spectral Karyotyping and FISH**

**Spectral karyotyping (SKY)** was performed to evaluate chromosome number (ploidy), and structural aberrations. 15-20 metaphase spreads were prepared for each cell line, hybridized with custom chromosome-specific fluorescence spectral karyotyping (SKY) probes, imaged, and analyzed using the SkyView software (Applied Spectral Imaging, California, CA), as previously described (3). Karyotype descriptions were produced in accordance with the human chromosome nomenclature standards described in ISCN (2013) (4). A structural aberration or chromosomal gain was considered clonal if two or more metaphase spreads contained the same change, while chromosomal losses were considered clonal if three or more metaphase spreads demonstrated the same loss (3, 4).

**Fluorescent in situ hybridization (FISH):** Break-apart FISH assays were performed to evaluate chromosomal translocations involving the *TFE3* locus. Serial 3-4μm thick paraffin sections of tissues were cut and mounted on positively charged glass slides, deparaffinized with xylene and ethanol, treated with pre-treatment solution (2×SSC with 0.5% NP-40) for 45 min at 85°C, followed by proteinase treatment (0.2% Pepsin in 0.001N HCL) for 20 minutes at 37°C. Upon dehydration with ethanol, tissues were denaturated (70% Formamide, 1×SSC, pH7.0) at 74°C for 5min. Incubation with FISH probes for TFE3 (chr Xp11.2; ZytoVision Z-2109) was performed at 75°C for 10 min followed by incubation at 37°C for 16 – 24h in a moisture chamber. Slides were then consecutively washed with 0.5xSSC+0.1%NP40 solution for 10min at 74°C, 2xSSC+0.1%NP40 at room temperature for 1 min, rinsed in ddH_2_O and air dried before mounting in mounting medium with DAPI. Slides were imaged with an AxioScan.Z1 Slide Scanner (Zeiss, Oberkochen, DE).

**In Vivo Studies**

Frederick National Laboratory for Cancer Research is accredited by AAALAC International and follows the Public Health Service Policy for the Care and Use of Laboratory Animals. Animal care was provided in accordance with the procedures outlined in the “Guide for the Care and Use of Laboratory Animal*s*” (National Research Council; 2011; National Academies Press; Washington, DC). All animal studies were approved by the Animal Care and Use Committee of the Frederick National Laboratory for Cancer Research.

Tumor xenografts were generated by injecting 5×10^6^ UOK124 or UOK146 cells subcutaneously into the right flank of 6-8 week old female athymic nude mice (Charles River) as cell suspensions in Matrigel (BD Biosciences, Franklin Lakes, NY). In approximately 2 weeks (UOK124) or 6 weeks (UOK146), tumors reached 50-150 mm^3^ in volume (from digital caliper measurements of length × width × height × pi/6). Mice were randomized into treatment groups (n = 10 mice/group) based on tumor volume using the Studylog software (Studylog Systems). Drug treatments were as follows: (1) NVP-BGT226, 5mg/kg or vehicle [% N-methyl-2-pyrrolidone (NMP)/90% PEG300], p.o., daily for 8 weeks; (2) Mithramycin A, 0.75mg/kg, or vehicle (phosphate-buffered saline), i.p., 3 x per week for 8 weeks; (3) Dasatinib, 25mg/kg or vehicle (4%DMSO, 30%PEG300, 5% Tween 80), p.o., daily for 8 weeks; (4) Carfilzomib, 2mg/kg or vehicle (10% sulfobutylether β-cyclodextrin in 10 mmol/L citrate buffer pH 3.5), i.v., 2 x per week for 8 weeks; (5) CDX-011, 2.5mg/kg or vehicle (phosphate-buffered saline), i.v., q4D for 4 doses. The same doses, mode of delivery and dosing frequency were used for combination studies. Studies were set up as follows: (1) all 5 drugs in xenografts from both cell lines; separate CDX-011 study; (2) 3 drugs (Dasatinib, BGT226, Mithramycin A) in xenografts from both cell lines; (3) combination studies in xenografts from both cell lines.

Tumor dimensions were monitored twice weekly by digital caliper during treatment and for up to one month after end of treatment. For the duration of the experiment, mice were monitored by observation of overall health and weekly body weights to determine drug tolerability. Mice were sacrificed at tumor endpoint when the longest tumor diameter reached 2 cm. Mouse survival was calculated as Log-rank test in Graphpad Prism. Tumor growth was calculated as a rate-based T/C metric according to Hather et al.,2014 (5) by fitting each tumor’s growth to an exponential model and calculating rate -based T/C as 10^(𝜇𝑇−𝜇𝑐)^ x (number of experiment days), where 𝜇𝑇 is the mean of the growth rates for the treatment group and 𝜇𝑐 is the mean growth rate of the control group. Values lower than 0.4 were considered to be significantly different (5).

**References:**

1. Mathews Griner LA, Guha R, Shinn P, Young RM, Keller JM, Liu D, et al. High-throughput combinatorial screening identifies drugs that cooperate with ibrutinib to kill activated B-cell-like diffuse large B-cell lymphoma cells. Proc Natl Acad Sci U S A. 2014;111(6):2349-54.

2. Lin GL, Wilson KM, Ceribelli M, Stanton BZ, Woo PJ, Kreimer S, et al. Therapeutic strategies for diffuse midline glioma from high-throughput combination drug screening. Sci Transl Med. 2019;11(519).

3. Padilla-Nash HM, Barenboim-Stapleton L, Difilippantonio MJ, Ried T. Spectral karyotyping analysis of human and mouse chromosomes. Nat Protoc. 2006;1(6):3129-42.

4. Simons A, Shaffer LG, Hastings RJ. Cytogenetic Nomenclature: Changes in the ISCN 2013 Compared to the 2009 Edition. Cytogenet Genome Res. 2013;141(1):1-6.

5. Hather G, Liu R, Bandi S, Mettetal J, Manfredi M, Shyu WC, et al. Growth rate analysis and efficient experimental design for tumor xenograft studies. Cancer Inform. 2014;13(Suppl 4):65-72.
